# SVCV phosphoprotein hijacks phase separation to immobilize the IRF3-TBK1 signaling axis and suppress interferon antiviral immunity

**DOI:** 10.1128/jvi.01387-25

**Published:** 2025-12-09

**Authors:** Yueyi Wang, Fengyun Wu, Dongdong Fan, Aifu Lin, Lixin Xiang, Ye Chen, Jianzhong Shao

**Affiliations:** 1 College of Life Sciences, Key Laboratory of Cell and Molecular Intelligent Design and Development of Zhejiang Province, Division of Medical Genetics and Genomics, the Children’s Hospital, Zhejiang University School of Medicine, Zhejiang University 12377, Hangzhou, China; 2 Laboratory for Marine Biology and Biotechnology, Qingdao Marine Science and Technology Center, Qingdao, China; University Medical Center Freiburg, Freiburg, Germany

**Keywords:** SVCV phosphoprotein, phase separation, interferon regulation, viral evasion, TBK1-IRF3 signaling axis

## Abstract

Spring viremia of carp virus (SVCV), a highly pathogenic rhabdovirus prevalent in fish, causes substantial mortality by evading host antiviral immunity; however, the underlying mechanisms remain incompletely understood. This study reveals a novel immune evasion strategy whereby the SVCV phosphoprotein (SVCV-P) hijacks the TBK1-IRF3 signaling axis via liquid-solid phase transition (LSPT), sequestering interferon regulatory factor 3 (IRF3) and inhibiting interferon (IFN) production. Upon stimulation, IRF3 facilitates TBK1 into functional liquid-liquid phase separation (LLPS) condensates, spatially enhancing IRF3 phosphorylation and downstream IFN responses. IRF3 acts as a scaffold via its DNA-binding domain (DBD) and intrinsically disordered region (IDR), while TBK1 incorporates via its kinase domain (KD), ubiquitin-like domain (ULD), and scaffold dimerization domain (SDD). Conversely, SVCV-P, driven by its IDRs and central domain (CD), undergoes robust LLPS, competitively recruiting TBK1 into SVCV-P-TBK1 condensates. These condensates merge with IRF3-TBK1 droplets, forming SVCV-P-TBK1-IRF3 ternary condensates. These subsequently undergo LSPT, immobilizing IRF3 and preventing its nuclear translocation. *In vitro* reconstitution and domain-deletion assays confirmed key domain roles in mediating LLPS and LSPT. Disrupting SVCV-P LLPS restored IFN expression and reduced viral replication *in vitro*. Zebrafish infection models demonstrated SVCV-P-mediated LLPS impaired IFN signaling and increased mortality. Phase-separation-deficient mutants (SVCV-P_ΔIDR_) lost immunosuppressive activity; this defect was rescued by chimeric SVCV-P proteins with heterologous LLPS domains. This study unravels a novel LLPS-dependent mechanism for TBK1-IRF3 signalosome regulation and demonstrates how SVCV hijacks phase separation to remodel host complexes into pathological aggregates, providing a paradigm for viral immune evasion and suggesting new antiviral targets.

**IMPORTANCE:**

Understanding interferon (IFN) signaling regulation and viral evasion is central to host-pathogen interactions. The discovery of liquid-liquid phase separation (LLPS) in cellular activities provides a new perspective for such investigations. Spring viremia of carp virus (SVCV), a severe fish pathogen, has potent IFN evasion capabilities, making it an attractive research model. Here, we demonstrate that LLPS spatially enhances IFN production by concentrating interferon regulatory factor 3 (IRF3) and TANK-binding kinase 1 (TBK1) into functional droplets, thereby boosting IRF3 activation. However, the SVCV phosphoprotein (SVCV-P) disrupts this via dual phase-separation mechanisms. First, SVCV-P undergoes LLPS to hijack TBK1 into viral-host condensates, sequestering it from IRF3. Second, these droplets merge with host defense droplets, trapping IRF3 within ternary aggregates. This paralyzes IRF3, blocking its nuclear translocation and IFN production. These findings provide new insights into how viruses exploit phase separation to block innate immune signaling, highlighting LLPS as a promising cross-species antiviral target.

## INTRODUCTION

Spring viremia of carp virus (SVCV), a member of the genus Sprivivirus within the family Rhabdoviridae, is a highly pathogenic agent that causes spring viremia of carp (SVC), a devastating disease severely threatening cyprinid and other fishes (1–3). Characterized by seasonal outbreaks and high mortality, SVC causes substantial economic losses in the global aquaculture industry and fish resources, with major reports in Europe, Asia, and North America (3). The SVCV genome comprises a single-stranded negative-sense RNA containing five genes that sequentially encode for nucleoprotein (N), phosphoprotein (P), matrix protein (M), glycoprotein (G), and RNA-dependent RNA polymerase (L) (4). The N protein associates with viral RNA to form a ribonucleoprotein complex, while the P protein interacts with N and L proteins to regulate genome transcription and replication. The M protein facilitates viral assembly and budding by mediating interactions between the N protein, membrane phospholipids, and the G protein, thereby connecting the nucleocapsid to the viral envelope. The G protein enables virus entry into host cells via receptor binding, and the L protein is a major RNA polymerase component for the replication of viral genomic RNA (3–8). These genomic and functional features align SVCV with conserved characteristics of other known rhabdoviruses, such as rabies virus (RABV) and vesicular stomatitis virus (VSV), two representative members of the rhabdovirus family (9, 10). Given the broad host range of rhabdoviruses, spanning vertebrates, invertebrates, and plants, they serve as pivotal models for comparative virology and evolutionary studies (9, 11). Notably, RABV and VSV have become archetypal systems for investigating the mechanisms underlying replication, infection, and pathogenesis of negative-strand RNA viruses (7, 12). In recent years, aquatic rhabdoviruses represented by SVCV have garnered increasing attention, with emerging studies revealing intricate virus-host interactions. For instance, SVCV infection disrupts host autophagy, apoptosis, and innate immune signaling, particularly interferon (IFN) pathways, to evade antiviral defenses (13, 14). These findings not only elucidate the intrinsic strategies of SVCV during host-pathogen interaction but also provide novel insights into rhabdovirus biology, positioning SVCV as a promising model rhabdovirus complementary to RABV and VSV for understanding principal mechanisms of immune evasion, thereby offering unique research value in virology.

IFNs constitute the first line of host innate immune defense against viral invasion. Upon RNA virus infection, intracellular pattern recognition receptors such as RIG-I detect viral RNA, triggering IFN-inducing signaling cascades (15, 16). Activated RIG-I recruits the mitochondrial adaptor protein MAVS, which aggregates to form signaling platforms that recruit TRAF3/6 and downstream kinases like TBK1. This cascade culminates in the phosphorylation and nuclear translocation of transcription factors interferon regulatory factor 3 (IRF3)/IRF7, driving IFN and pro-inflammatory cytokine expression (17–19). To counteract this defense, RNA viruses have evolved hierarchical strategies to disrupt the RIG-I/MAVS signaling axis. For example, SARS-CoV suppresses IFN production by inhibiting MAVS/TRAF3/6 signalosome activity on mitochondria via its ORF-9b protein (20). SARS-CoV-2 disrupts MAVS aggregation through its membrane glycoprotein, thereby blocking TRAF3/TBK1 recruitment and subsequent IRF3 phosphorylation (21). Kaposi’s sarcoma-associated herpesvirus tegument protein ORF33 attenuates IFN-inducing signaling pathway by promoting MAVS dephosphorylation (22). Foot-and-mouth disease virus leader proteinase (Lbpro) antagonizes IFN-mediated innate immune responses by suppressing TRAF3 ubiquitination (23). Respiratory syncytial virus targets TRAF3 for degradation through NS1/NS2 proteins, forming a degradosome complex to suppress MAVS signaling pathways (24, 25). Recent studies demonstrate that SVCV employs multiple strategies to subvert the RIG-I/MAVS signaling axis for IFN immune evasion. For instance, the M protein prevents K63-linked TRAF3 ubiquitination by competing for binding sites with MAVS (26). The N protein promotes ubiquitin-dependent degradation of MAVS and viperin_sv1 (27, 28), and the P protein acts as a decoy substrate to inhibit IRF3 phosphorylation by competing for TBK1’s kinase domain (KD) (29). These findings highlight that distinct viral proteins can suppress the IFN-inducing signaling pathway by selectively targeting specific components of the RIG-I/MAVS signaling axis. However, the precise spatiotemporal regulation of these hierarchical interventions has yet to be clarified.

Intracellular signaling cascades rely on precise spatiotemporal dynamics, involving subcellular localization, sequential docking, and stepwise activation of pathway proteins (30–32). In recent years, biomolecular liquid-liquid phase separation (LLPS) has emerged as a pivotal mechanism for achieving highly regulated hierarchical control in diverse cellular activities, such as signal transduction (33), metabolic control (34), and structural assembly (35). This positions LLPS at the cutting edge of biological research. Notably, innate immune pathways leading to IFN production, exemplified by RIG-I/MAVS and cGAS/STING axes, have been shown to employ LLPS for regulatory control. For instance, RIG-I undergoes LLPS via intermolecular disulfide bonds to promote MAVS aggregation and activation, while TOLLIP modulates MAVS SUMOylation via phase separation to suppress MAVS aggregation and downstream signaling (36, 37). Additionally, IRF3, activated by the RIG-I/MAVS cascade, condensates with the promoter DNA of IFN genes in the nucleus to enhance gene expression (38). These discoveries advanced our understanding of spatiotemporal regulation in host IFN-inducing pathways and opened new avenues for investigating viral immune-evasion strategies that hijack LLPS. Here, we report that IRF3 drives TBK1 into functional condensates via LLPS, enriching their interaction spatially to enhance IRF3 phosphorylation while preventing crosstalk with other pathways. This reveals a novel LLPS-mediated regulatory mechanism for the TBK1-IRF3 signaling axis, a central hub of the RIG-I/MAVS pathway. Furthermore, SVCV phosphoprotein (SVCV-P) exhibits robust LLPS activity and competitively recruits TBK1 into SVCV-P-TBK1 condensates, reducing IRF3 incorporation into IRF3-TBK1 condensates. Critically, SVCV-P-TBK1 condensates merge with IRF3-TBK1 droplets to form solidified ternary SVCV-P-TBK1-IRF3 aggregates. This process immobilizes IRF3, preventing its nuclear translocation and IFN gene activation. Using *in vitro* reconstitution, we mapped the domains of IRF3, SVCV-P, and TBK1 responsible for driving LLPS and liquid-solid phase transition (LSPT) in these aggregates. Functional validation, both *in vitro* and *in vivo*, confirmed that disrupting SVCV-P LLPS restored IFN expression and reduced viral replication. Our findings establish a paradigm wherein SVCV exploits phase separation to hijack TBK1-IRF3 signaling, thereby subverting host IFN-mediated immunity. Given the conservation of phase separation mechanisms across species, these findings illuminate a broader viral immune evasion strategy employed by rhabdoviruses, with implications extending to mammalian pathogens.

## RESULTS

### Phase separation of IRF3-TBK1 in the IFN-inducing signaling pathway

**Fig 1:**
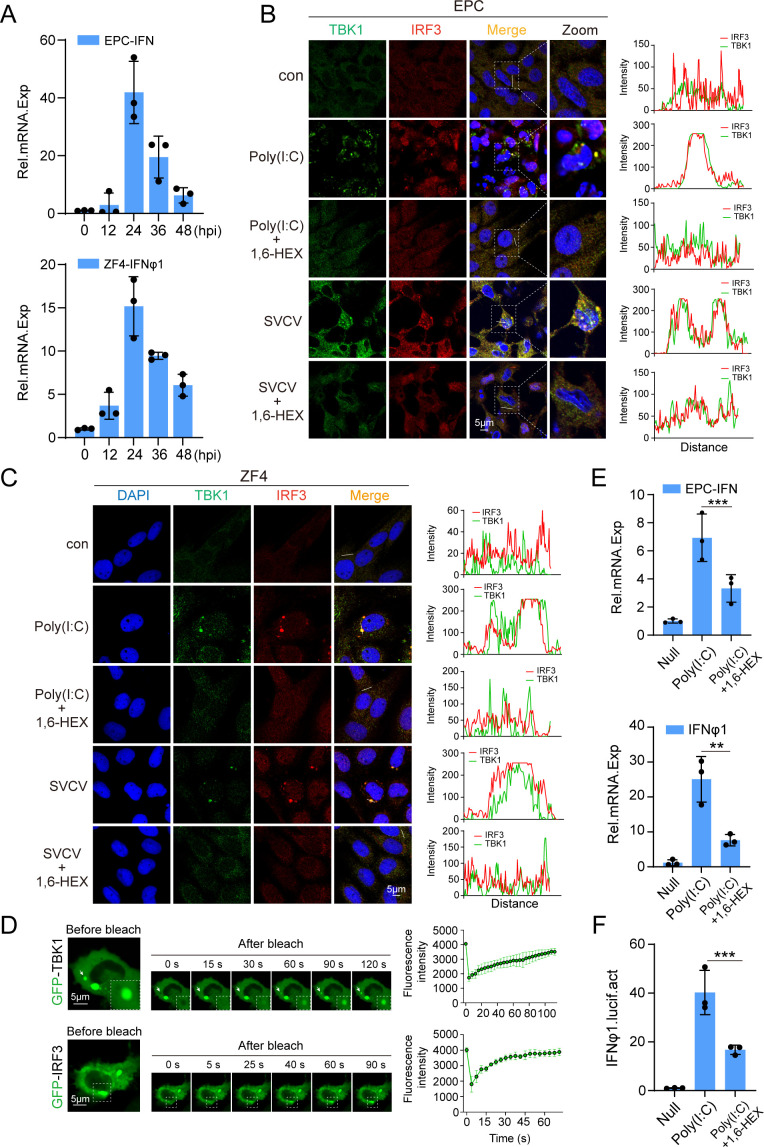
LLPS of the IRF3-TBK1 complex in IFN-inducing signaling. (**A**) Inducible expression patterns of IFNs in EPC and ZF4 cells following poly(I:C) treatment. Cells were seeded on six-well plates overnight and treated with poly(I:C) (10 µg/mL). Total RNAs were extracted at the indicated time points (0, 12, 24, 36, and 48 h) for RT-qPCR analysis. (**B and C**) Illustration of the co-localization of IRF3 and TBK1 in EPC and ZF4 cells using IF staining. Cells were infected with 100 TCID_50_ of SVCV (5 × 10^6^ TCID_50_/mL) or treated with poly(I:C) (10 µg/mL) for 24 h, followed by mock treatment or treatment with 1,6-HEX for 1 min before staining with anti-IRF3 and anti-TBK1 antibodies. Intensity profiles of the indicated proteins along quantitative lines are shown in the right panels, as analyzed by ImageJ line scan analysis. Images were captured under a confocal laser scanning microscope (Olympus FV3000, original magnification, ×630). Scale bar: 5 µm. (**D**) Representative micrographs of GFP-TBK1 and GFP-IRF3 puncta FRAP in EPC cells (left) and their quantifications (right). (**E**) The transcription of IFNs induced by poly(I:C) was inhibited by treatment with 1,6-HEX in EPC or ZF4 cells. Cells were exposed to poly(I:C) (10 µg/mL) for 12 h, then either mock treated or treated with 1,6-HEX for 1 min before extracting RNA for RT-qPCR analysis. (**F**) Effect of 1,6-HEX on the poly(I:C)-induced activation of the IFNφ1 promoter. EPC cells were transfected with 250 ng of IFNφ1-pro and 25 ng of pRL-TK reporter plasmids. After 24 h, cells were treated with poly(I:C) (10 µg/mL), followed by mock treatment or treatment with 1,6-HEX for 1 min. Luciferase activities were then monitored after stimulation. Data represent means ± SD from three independent experiments (^**^P < 0.01; ^***^P < 0.001; NS, not significant).

Previous research shows that after activation in the cytoplasm, IRF3 translocates to the nucleus and interacts with IFN gene promoters via LLPS, thereby initiating IFN expression (38). This demonstrates an LLPS-dependent mechanism regulating IRF3’s transcriptional activity. However, it remains unclear whether IRF3 activation depends on LLPS during cytoplasmic IFN-inducing signaling. To address this, we observed the phase separation activity of IRF3 and TBK1 in EPC and ZF4 cell lines by activating endogenous IFN-inducing signaling pathway through stimulating cells with poly(I:C). RT-qPCR analysis revealed a significant increase in IFN gene expression in both EPC and ZF4 cells 12–24 h after poly(I:C) stimulation (Fig. 1A). Concurrently, immunofluorescence (IF) staining using anti-IRF3 and anti-TBK1 antibodies demonstrated the formation of endogenous IRF3-TBK1 co-condensates in the cytoplasm of EPC and ZF4 cells upon poly(I:C) stimulation (Fig. 1B and C). Fluorescence recovery after photobleaching (FRAP) experiments indicated that IRF3 and TBK1 in the co-condensates exhibited fluorescence recovery, consistent with the highly dynamic nature of liquid-liquid phase-separated droplets (Fig. 1D). Furthermore, treatment with the phase separation inhibitor 1,6-hexanediol (1,6-HEX) significantly reduced the number of poly(I:C)-induced IRF3-TBK1 co-condensate droplets (Fig. 1B and C), downregulated IFN gene expression in EPC and ZF4 cells (Fig. 1E), and inhibited the activity of the IFNφ1-pro fluorescent reporter in EPC cells (Fig. 1F). Additionally, the IRF3-TBK1 co-condensates were also observed in the cytoplasm of SVCV-infected EPC and ZF4 cells, and 1,6-HEX treatment markedly inhibited the formation of SVCV-induced co-condensates in both of the cell lines (Fig. 1B and C). These findings collectively suggested the induction of IRF3-TBK1 co-condensates through a phase-separation pathway under stimulation of poly(I:C) and SVCV, and the phase separation of IRF3-TBK1 is critical for activating the IFN-inducing signaling pathway.

### IRF3 acts as a scaffold protein in driving IRF3-TBK1 phase separation

**Fig 2:**
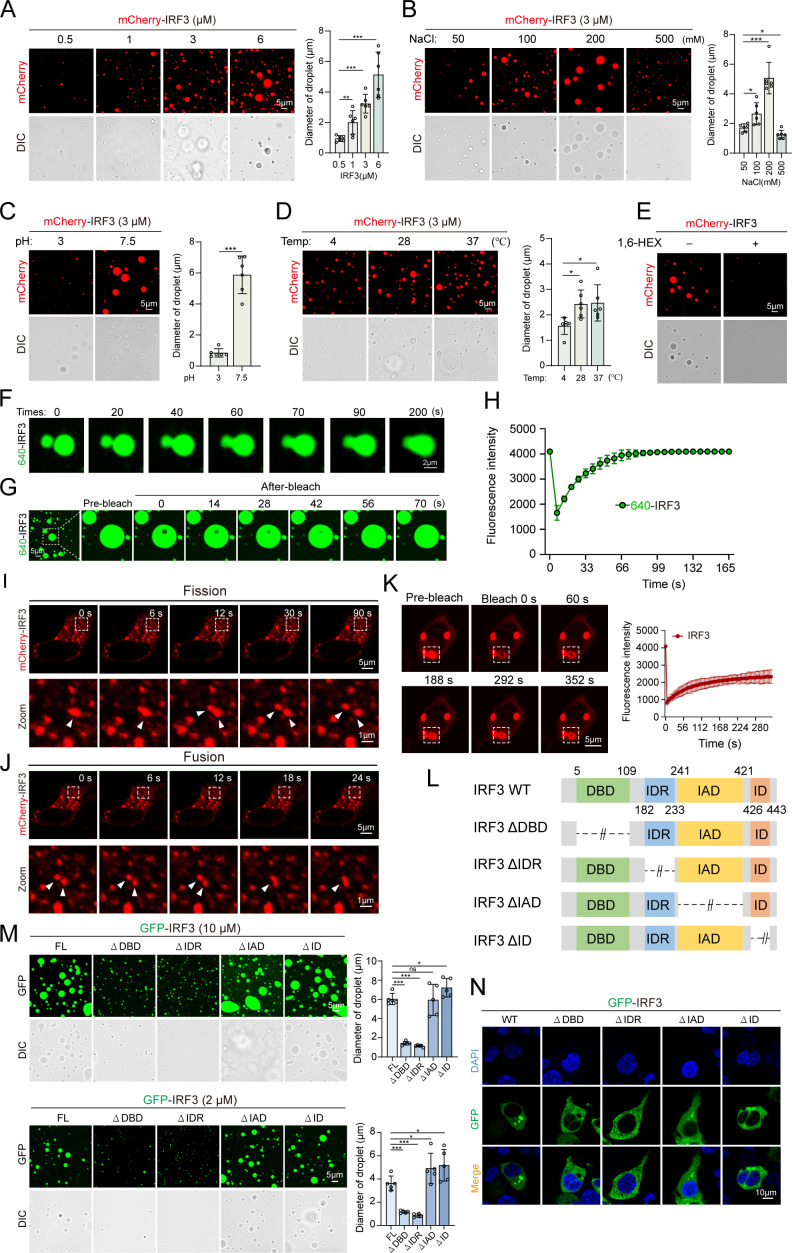
LLPS of IRF3. (**A–D**) Images of GFP-IRF3 droplet formation at room temperature with varying GFP-IRF3 concentrations, NaCl gradients, pH values, and temperatures. Images were captured under a confocal laser scanning microscope (Olympus FV3000, original magnification, ×630). Right, quantitative analyses of GFP-IRF3 droplet diameters at different concentrations (**A**), NaCl gradients (**B**), pH values (**C**), and temperatures (**D**), *n*  =  6 for each experiment. DIC: differential interference contrast. Scale bar: 5 µm. (**E**) GFP-IRF3 proteins (3 µM) treated with 5% 1,6-HEX *in vitro*. (**F**) Representative images showing fusion of 640-IRF3 droplets *in vitro* at a concentration of 5 µM in 200 mM NaCl. Scale bar: 2 µm. (**G, H**) Representative FRAP of 640-IRF3 droplets *in vitro* at the concentrations of 5 µM in 200 mM NaCl and quantification of 640-IRF3 droplets over a 170-s time course. Scale bar: 5 µm. (**I, J**) Fusion and fission of mCherry-IRF3 puncta in HEK293T cells transfected with pmCherry-IRF3. After 24 h, live cells were analyzed, and images were captured under a confocal laser scanning microscope (Olympus FV3000, original magnification, ×630). Scale bar: 5 µm. (**K**) Representative images of GFP-IRF3 puncta FRAP *in vivo* (left) and corresponding quantification (right). Scale bar: 5 µm. (**L**) Depiction of the IRF3 protein variants. (**M**) Representative micrographs of GFP-IRF3 and its four IRF3 variants (ΔDBD, ΔIDR, ΔIAD, and ΔID) forming droplets at two concentrations (10 µM and 2 µM) *in vitro*. Right, quantitative analyses of droplet diameters for GFP-IRF3 and its four variants, *n* = 5 for each experiment. Scale bar: 5 µm. (**N**) Subcellular localization of GFP-IRF3 and its four IRF3 variants (ΔDBD, ΔIDR, ΔIAD, and ΔID). Data are representative of three independent experiments and are presented as means ± SD (^*^P < 0.05; ^**^P < 0.01; ^***^P < 0.001; NS, not significant).

As IRF3 contains an N-terminal intrinsically disordered region (IDR), a domain known as essential and sufficient for LLPS, we hypothesized that IRF3 may act as a scaffold molecule with driving capability for IRF3-TBK1 co-condensate formation. To provide evidence, the zebrafish IRF3 and TBK1 proteins were selectively used as a model system throughout our research. We first expressed and purified the mCherry-IRF3 fusion protein from *E. coli* for an *in vitro* LLPS assay. As expected, the mCherry-IRF3 protein was capable of condensing into spherical droplets (Fig. 2A). Moreover, the abundance of mCherry-IRF3 droplets increased markedly, transitioning from barely noticeable small foci to well-defined droplets and large droplet clusters, as protein and salt concentrations, pH, and temperature gradients increased (Fig. 2A through D). Notably, elevated salt concentration (500 mM) and temperature (37°C) did not further enhance droplet abundance but instead exhibited inhibitory effects, indicating efficient condensation of IRF3 liquid droplets at physiological conditions. Correspondingly, the addition of 5% 1,6-HEX significantly inhibited the formation of IRF3 droplets (Fig. 2E). Additionally, we expressed and purified only His-tagged IRF3, labeled it with the Alexa Fluor 640 dye, and observed that two approaching AF640-labeled IRF3 droplets fused into a larger droplet (Fig. 2F), ruling out the possibility that the LLPS puncta were artificially formed by the mCherry tag. Furthermore, after selective FRAP of the droplet’s central region, rapid fluorescence recovery was observed (Fig. 2G and H), indicating potential rapid redistribution of IRF3 protein between the liquid droplets and surrounding cellular proteins. We next characterized IRF3 puncta in HEK293T cells transfected with mCherry-IRF3 using live imaging and FRAP assays. The results showed that mCherry-IRF3 puncta underwent fusion and fission processes and rapid fluorescence recovery, demonstrating highly dynamic activity (Fig. 2I through K). To determine which domain of IRF3 is responsible for its phase separation, we expressed and purified a series of deletion mutants (IRF3-∆DBD, ∆IDR, ∆IAD, ∆ID) devoid of the DNA-binding domain (DBD), IDR, IRF association domain (IAD), and inhibitory domain (ID), each tagged with EGFP, in both HEK293T and *E. coli* cells (Fig. 2L). We compared the droplet sizes of these mutant proteins at two concentrations (2 µM and 10 µM) to assess *in vitro* phase separation. The results showed that deletion of the DBD and IDR domains significantly decreased IRF3 droplet formation (Fig. 2M). However, mutants lacking the IAD and the ID domains formed droplets that were similar or slightly larger in size compared to those of wild-type IRF3 (Fig. 2M). Consistent with these *in vitro* results, overexpressed IRF3 mutants lacking the DBD and IDR domains did not form phase-separated droplets in HEK293T cells (Fig. 2N). These findings indicate that IRF3 forms phase-separated droplets both *in vitro* and *in vivo*, with the DBD and IDR domains being crucial in this process.

### TBK1 acts as a client protein in IRF3-driven phase separation

**Fig 3:**
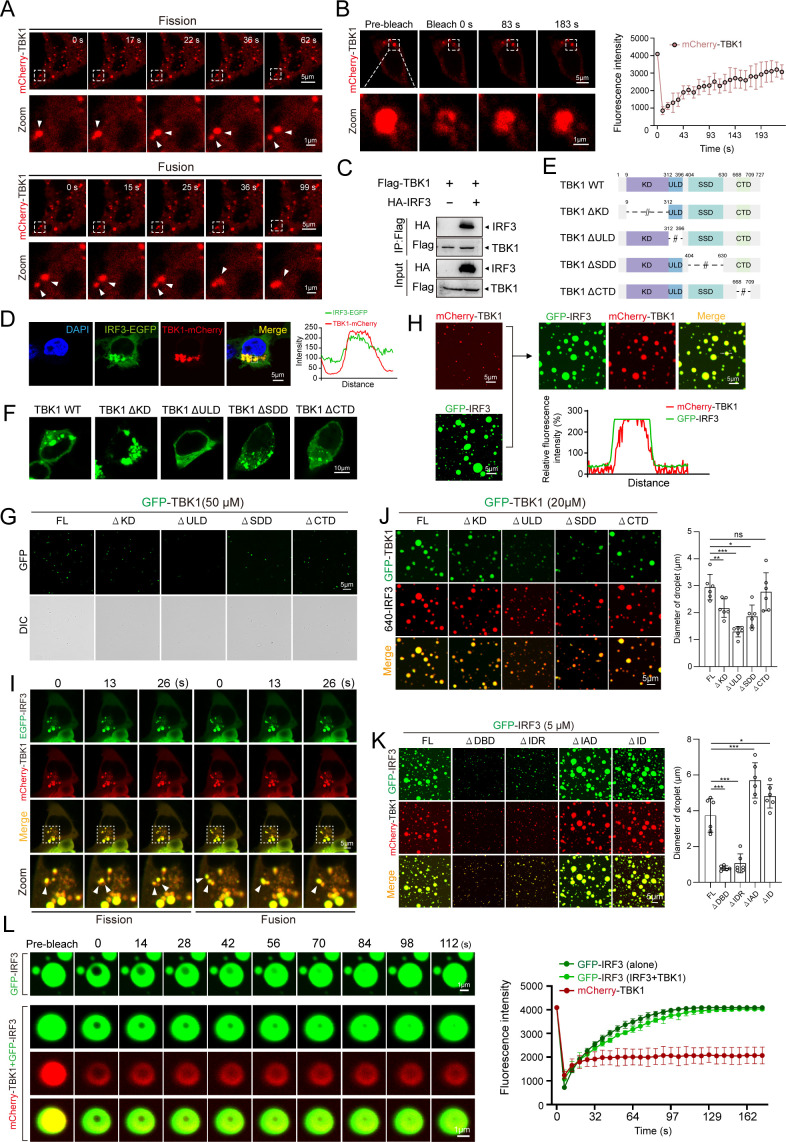
Illustration of TBK1’s role as a client protein in the IRF3-driven phase separation process. (**A**) Fusion (bottom panel) and fission (top panel) dynamics of mCherry-TBK1 puncta were observed in HEK293T cells transfected with pmCherry-TBK1. After 24 h, live cells were analyzed, and images were captured under a confocal laser scanning microscope (Olympus FV3000, original magnification, ×630). Scale bar: 5 µm. (**B**) Representative images showing FRAP of mCherry-TBK1 puncta *in vivo* (left) and quantified fluorescence recovery intensity over a 200-s time course (right). Scale bar: 5 µm. (**C**) Co-IP of TBK1 and IRF3. HEK293T cells transfected with specified plasmids underwent immunoprecipitation (IP) with anti-Flag antibody, followed by Western blotting using anti-HA antibody to detect the interaction. (**D**) Analysis of IRF3 and TBK1 co-localization. HEK293T cells were transfected for 24 h with plasmids encoding GFP-IRF3 and mCherry-TBK1, then fixed and examined by confocal microscopy. The right panels show the intensity profiles of the indicated proteins along the plotted lines, as determined by ImageJ line scan analysis. Images were captured under a confocal laser scanning microscope (Olympus FV3000, original magnification, ×630). Scale bar: 5 µm. (**E**) Schematic of TBK1 protein variants. (**F**) Subcellular localization of GFP-TBK1 and four TBK1 variants (ΔKD, ΔULD, ΔSDD, and ΔCTD) in HEK293T cells. (**G**) Representative images of *in vitro* droplet formation of GFP-TBK1 and four TBK1 variants (ΔKD, ΔULD, ΔSDD, and ΔCTD). Scale bar: 5 µm. (**H**) Coalescence of preformed GFP-IRF3 and mCherry-TBK1 droplets into biphasic condensates *in vitro*. Fluorescence line profiles (bottom) were generated using ImageJ line scan analysis. (**I**) Fission (left) and fusion (right) dynamics of co-localized GFP-IRF3/mCherry-TBK1 condensates in HEK293T cells. Images show indicated time points after imaging initiation. Scale bar: 5 µm. (**J and K**) Fluorescence microscopy images of TBK1 mutants with IRF3 and IRF3 mutants with TBK1. GFP-TBK1 (FL or mutants) with 640-IRF3 and GFP-IRF3 (FL or mutants) with mCherry-TBK1 were co-incubated and imaged for mixture droplet formation. Scale bar: 5 µm. Right, quantitative analysis of droplet diameters for the mixture droplet (*n* = 6 for each experiment, ^*^P < 0.05; ^**^P < 0.01; ^***^P < 0.001). (**L**) Representative images of GFP-IRF3 FRAP in the presence or absence of mCherry-TBK1 (left) and quantification (right) of over a 200-s time course (*n*  =  3 droplets). Images were captured under a confocal laser scanning microscope (Olympus FV3000, original magnification, ×630). Scale bar: 1 µm.

Upon receiving IFN-inducing signals, endogenous TBK1 and IRF3 form co-condensate droplets in stimulated cells. This observation suggests that TBK1 may have an intrinsic ability to undergo LLPS with the help of additional cellular components, including IRF3 in the IFN-inducing signaling pathway, since TBK1 lacks IDRs that are essential for liquid condensate formation. Consistent with this hypothesis, overexpressed mCherry-TBK1 forms droplets in HEK293T cells that undergo fusion and fission processes (Fig. 3A) and display highly dynamic behavior, confirmed by FRAP assay (Fig. 3B). However, minimal phase-separated droplets of mCherry-TBK1 or EGFP-TBK1 alone were observed in an *in vitro* assay under the same conditions for IRF3 (Fig. 3G). These findings indicate that TBK1 may function as a client protein, requiring interaction with the IRF3 scaffold protein to undergo LLPS *in vivo* due to the well-documented association between TBK1 and IRF3 within the IFN-inducing signaling pathway. Consistently, co-immunoprecipitation (Co-IP) confirmed the direct interaction between TBK1 and IRF3, while confocal imaging showed that these two proteins colocalize in phase-separated droplets in HEK293T cells (Fig. 3C and D). For further clarification, we expressed and purified mCherry-tagged TBK1 protein to assess its *in vitro* co-condensation with IRF3. As anticipated, when EGFP-IRF3 was co-incubated with mCherry-TBK1, it resulted in the formation of co-condensate droplets similar in size to those formed by EGFP-IRF3 alone. In contrast, mCherry-TBK1 exhibited weak phase separation capabilities when incubated alone (Fig. 3H). These findings confirm that TBK1 acts as a client protein, which can be recruited into the phase-separated droplets driven by the IRF3 scaffold protein. Notably, time-lapse imaging demonstrated dynamic fusion and fission events between IRF3-TBK1 co-condensates in HEK293T cells (Fig. 3I), indicative of LLPS behavior of these biphasic condensates.

To identify the domain of TBK1 that participates in concerted phase separation with IRF3, we generated a series of TBK1 mutants (TBK1-∆KD, ∆ULD, ∆SDD, ∆CTD) devoid of the N-terminal KD, ubiquitin-like domain (ULD), α-helical scaffold dimerization domain (SDD), and C-terminal adaptor-binding domain (CTD) (Fig. 3E). We evaluated the phase separation potential of these mutants both *in vitro* and *in vivo*. Most TBK1 mutants failed to form large phase-separated droplets *in vitro* (Fig. 3G), but mutants lacking the KD, SDD, and CTD domains still exhibited some phase separation activity in cells (Fig. 3F). Co-incubation of EGFP-tagged TBK1 mutants with IRF3 labeled with 640, or EGFP-tagged IRF3 mutants with mCherry-tagged TBK1, revealed that deletion of KD, ULD, and SDD domains of TBK1 or DBD and IDR domains of IRF3 severely impaired the formation of IRF3-TBK1 co-condensate droplets, indicating the contribution of these domains to the co-phase separation between TBK1 and IRF3 (Fig. 3J and K). Since TBK1 phosphorylates IRF3 and then induces its nuclear translocation upon IFN-inducing signaling, we propose that co-phase separation facilitates IRF3 phosphorylation and subsequent nuclear translocation from the condensates. We next assessed the dynamic activity of IRF3 within the IRF3-TBK1 condensates. FRAP analysis showed rapid fluorescence recovery of EGFP-IRF3 within the co-condensates after photobleaching, comparable to controls formed only by the EGFP-IRF3 (Fig. 3L). This finding suggests that co-phase separation with TBK1 did not significantly alter IRF3’s inherent LLPS ability (Fig. 3L). In contrast, mCherry-TBK1 within the co-condensates exhibited slow FRAP (Fig. 3L). It suggests that TBK1’s phase separation ability is weaker within the co-condensed IRF3-TBK1 compared to IRF3 alone (Fig. 3L). These results collectively indicated that IRF3 acts as a scaffold protein in driving the co-phase separation of TBK1 client protein, thereby compartmentalizing TBK1 in the IRF3-TBK1 co-condensates. Thus, the IRF3-TBK1 co-condensates may act as signalosomes facilitating IFN-inducing signaling, with multiple domain-domain interactions involved in the formation of this signalosome complex.

### Phase-separation activity of SVCV-P and its association with TBK1

**Fig 4:**
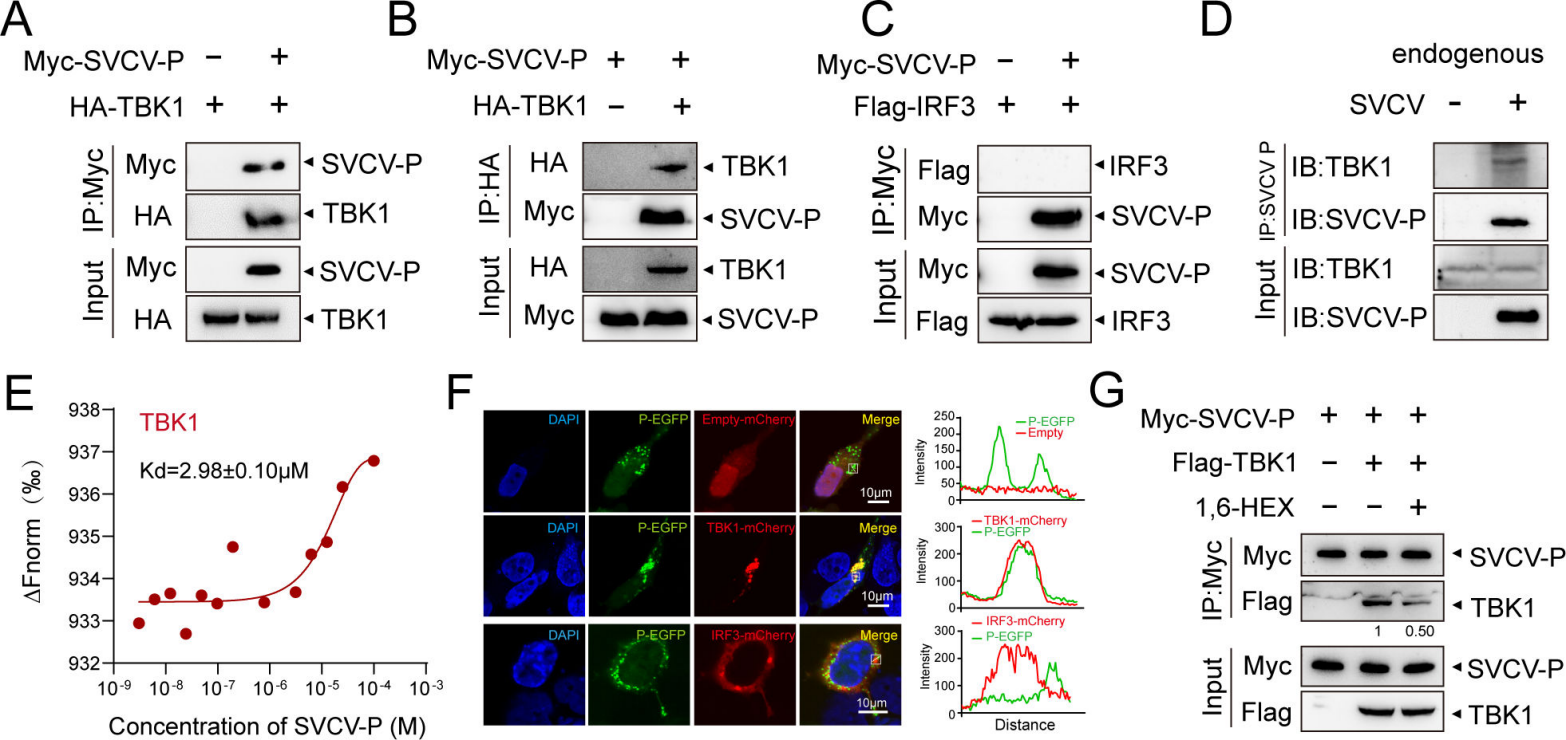
Interactions between SVCV-P, TBK1, and IRF3. (**A–C**) HEK293T cells were transfected with the indicated plasmids, and cell lysates were immunoprecipitated with anti-Myc/anti-HA antibodies and analyzed by Western blotting with corresponding antibodies. (**D**) Endogenous interaction between SVCV-P and TBK1. SVCV-infected EPC cells were subjected to IP using anti-SVCV-P antibody and analyzed by Western blotting using the anti-TBK1 antibody. (**E**) Measurement of the dissociation constant (*K*_d_) of SVCV-P for TBK1 by a MST assay. (**F**) Confocal microscopy images showing the co-localization of EGFP-SVCV-P with mCherry-TBK1 or mCherry-IRF3. HEK293T cells expressing EGFP-SVCV-P, mCherry-TBK1, or mCherry-IRF3 for 24 h, then the cells were fixed and imaged under a confocal laser scanning microscope (Olympus FV3000; magnification, ×630). Right panels show the intensity profiles of the indicated proteins along the plotted lines, as determined by ImageJ line scan analysis. Images were captured under a confocal laser scanning microscope (Olympus FV3000, original magnification, ×630). Scale bar: 10 µm. (**G**) Analysis of the interaction between SVCV-P and TBK1 under 1,6-HEX treatment. HEK293T cells were transfected with plasmids encoding Myc-SVCV-P and Flag-TBK1 for 36 h. Subsequently, the cells were treated with 1,6-HEX. The cell lysates were then immunoprecipitated using anti-Myc antibodies and analyzed by western blotting with corresponding antibodies.

**Fig 5:**
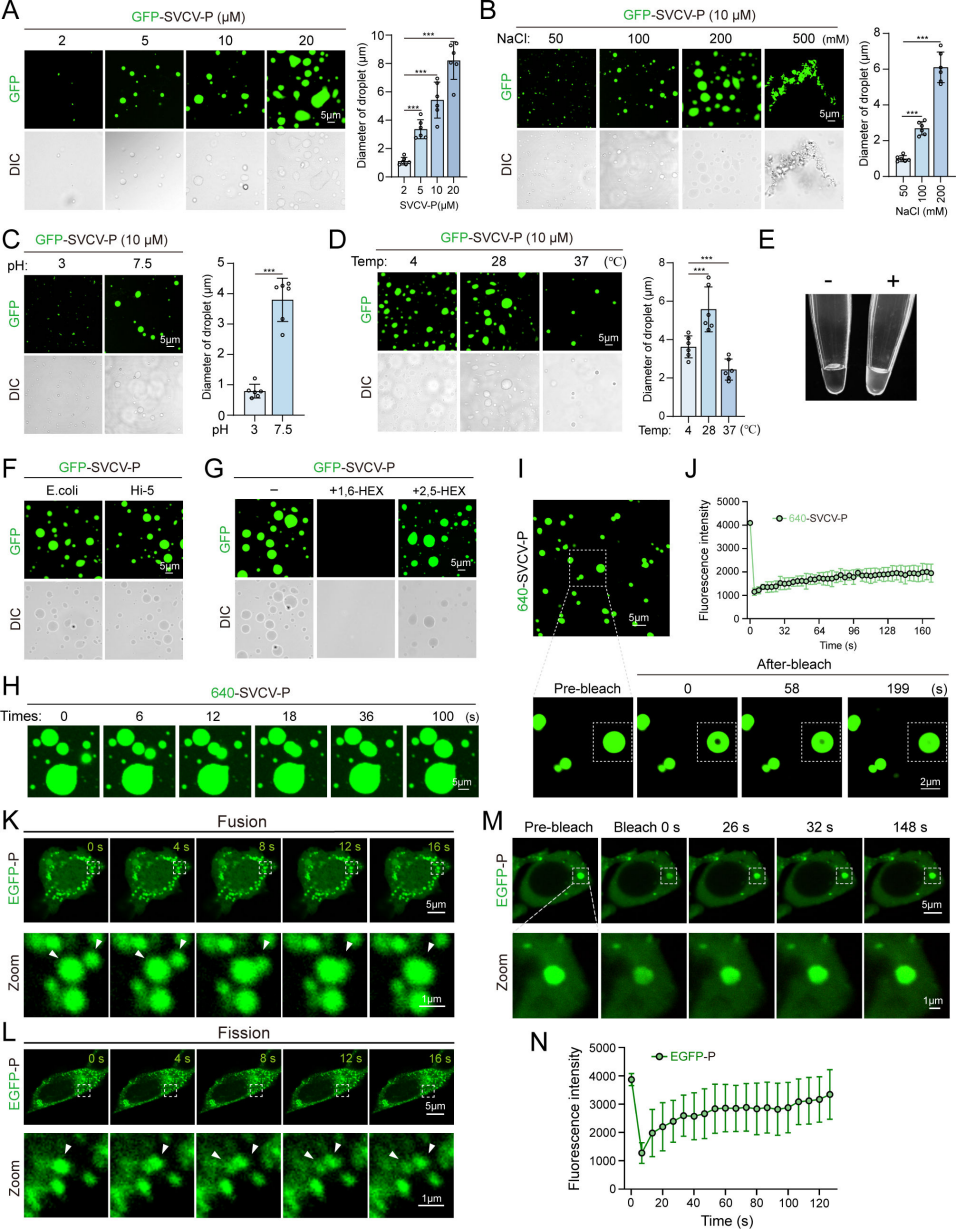
LLPS of SVCV-P. (**A–D**) Images of GFP-SVCV-P droplets at varying concentrations (**A**), NaCl gradients (**B**), pH values (**C**), and temperatures (**D**). Right, quantitative analysis of GFP-SVCV-P droplet diameters under these conditions, with *n*  =  6 for each experiment. DIC, differential interference contrast. Scale bar: 5 µm. (**E**) Turbidity of GFP-SVCV-P phase separation. Representative images are shown for GFP-SVCV-P solutions in the absence (−) or presence (+) of phase-separation buffer. Turbidity indicates the presence of phase-separated droplets. (**F**) Expression of GFP-SVCV-P protein from *E. coli* and Hi-5 cells. Representative fluorescence and DIC images are shown. Scale bar: 5 µm. (**G**) Effects of 5% 1,6-HEX or 5% 2,5-HEX on GFP-SVCV-P droplet formation *in vitro*. Scale bar: 5 µm. (**H**) Representative images showing fusion of GFP-SVCV-P droplets *in vitro*. Scale bar: 5 µm. (**I**) Representative FRAP of 640-SVCV-P droplets over a 200-s time course *in vitro*. (**J**) Quantitative analysis of FRAP recovery curve of 640-SVCV-P droplets. Scale bar: 5 µm. (**K and L**) Fusion and fission between GFP-SVCV-P puncta. HEK293T cells were transfected with pEGFP-SVCV-P. After 24 h, live cells were examined, and images were captured under a confocal laser scanning microscope (Olympus FV3000, original magnification, ×630). Scale bar: 5 µm. (**M and N**) Representative images of GFP-SVCV-P puncta FRAP *in vivo* (top) and quantification of GFP-SVCV-P puncta recovery (bottom). Images were captured under a confocal laser scanning microscope (Olympus FV3000, original magnification, ×630). Scale bar: 5 µm. Data are representative of three independent experiments and are shown as means ± SD (^*^P < 0.05; ^**^P < 0.01; ^***^P < 0.001; NS, not significant).

**Fig 6:**
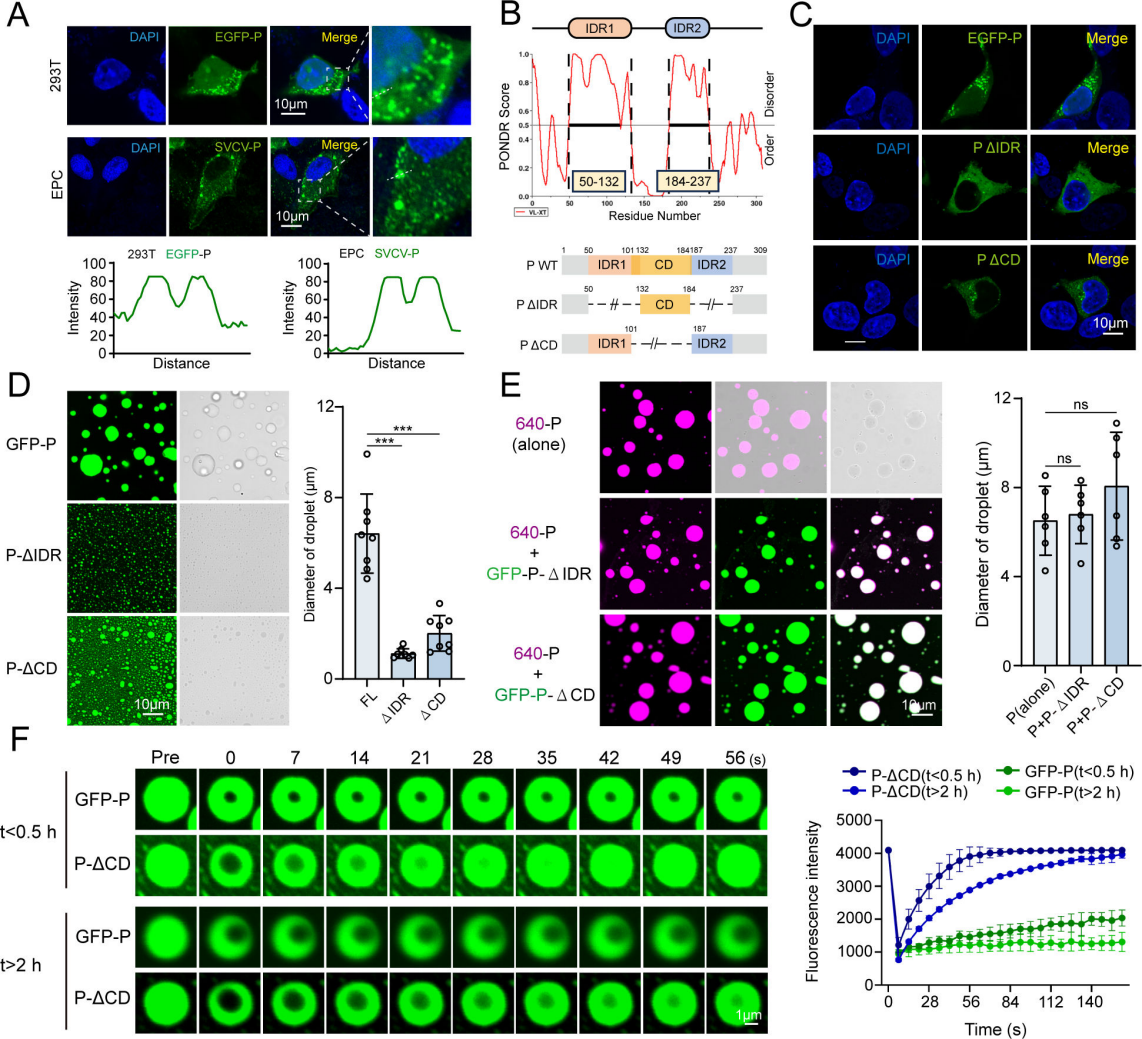
Functional domains of SVCV-P in LLPS. (**A**) Subcellular localization of SVCV-P in HEK293T cells with EGFP-SVCV-P overexpression or in SVCV-infected EPC cells. EPC cells were infected with 100 TCID_50_ of SVCV (5 × 10^6^ TCID_50_/mL), and HEK293T cells were transfected with an EGFP-SVCV-P encoding plasmid for 24 h. SVCV-P localization in EPC cells was detected using an anti-SVCV-P antibody and confocal microscopy (Olympus FV3000; ×630 original magnification). The bottom panels show the intensity profiles of the indicated proteins along the plotted lines, as analyzed by ImageJ line scan analysis. Scale bar: 10 µm. (**B**) Intrinsic disorder prediction (top) and schematic of truncated variants (bottom) of SVCV-P. (**C**) Subcellular localization of EGFP-SVCV-P, EGFP-SVCV-P-ΔIDR, and EGFP-SVCV-P-ΔCD in HEK293T cells. (**D**) Representative images of droplets formed by EGFP-SVCV-P and its variants (ΔIDR, ΔCD). Right, quantitative analysis of droplet diameters (*n* = 8 per group). Scale bar: 10 µm. (**E**) Fluorescence microscopy images of EGFP-SVCV-P-ΔIDR and GFP-SVCV-P-ΔCD co-incubated with 640-SVCV-P protein. Right, quantitative analysis of mixed droplet diameters (*n* = 6 per group). Scale bar: 10 µm. (**F**) Representative FRAP images (left) and recovery quantification (right) for EGFP-SVCV-P and EGFP-SVCV-P-ΔCD droplets over 200 s (*n* = 3). Images were captured by a confocal laser scanning microscope (Olympus FV3000, original magnification, ×630). Scale bar: 1 µm. Data are representative of three independent experiments and are shown as means ± SD (^***^P < 0.001; NS, not significant).

Previous studies have suggested that the IFN-inducing signaling pathway can be inhibited by the SVCV-P protein through disruption of the interaction between IRF3 and TBK1, potentially by competing with IRF3 for binding to TBK1 (29). However, the precise mechanisms of molecular interactions between SVCV-P, TBK1, and IRF3, as well as pathways underlying SVCV-P’s inhibition of TBK1 and IRF3 signaling, remain largely unclear. By bioinformatics prediction (IUpred2A, PONDR), two IDRs were identified in the SVCV-P protein, suggesting SVCV-P has a strong LLPS capability (Fig. 6B). This prompts us to hypothesize the potential role of SVCV-P in inhibiting IFN-inducing signaling pathway by the LLPS mechanism. To clarify this hypothesis, Co-IP and co-localization experiments were first conducted to assess the interactions between SVCV-P and TBK1 or IRF3 in HEK293T cells ectopically overexpressed with these proteins. The results revealed that SVCV-P interacts with TBK1 but does not bind IRF3 (Fig. 4A through C and F). This interaction was subsequently confirmed in SVCV-infected EPC cells, in which the endogenous viral P-protein associates with cellular TBK1 during SVCV infection (Fig. 4D). Additionally, microscale thermophoresis (MST) analysis demonstrated a direct affinity between SVCV-P and TBK1, with an equilibrium dissociation constant (*K*d) of 2.98 ± 0.10 µM, providing quantitative support for their interaction (Fig. 4E). Notably, treatment with 1,6-HEX weakened the interaction between SVCV-P and TBK1, indicating that LLPS facilitates this interaction (Fig. 4G). We next expressed and purified EGFP-tagged SVCV-P protein from *E. coli* cells for *in vitro* examinations. As expected, SVCV-P exhibited strong phase separation activity *in vitro*, with increased formation of droplets with increasing protein concentrations (5–20 μM) (Fig. 5A). The droplets became more numerous and larger with the increase of salt conditions (50–200 μM), with optimal phase separation occurring at a pH of 7.5 and a temperature of 28°C (Fig. 5B through D). Notably, high salt concentration (500 µM) promoted the precipitation of SVCV-P (Fig. 5B), while adding the protein to phase separation buffers led to visible turbidity (Fig. 5E), further indicating LLPS behavior of SVCV-P. To exclude the potential impact of phosphorylation modifications, we also purified SVCV-P using a recombinant baculovirus in Hi-5 lepidoptera cells. Both eukaryotic and prokaryotic expressions of SVCV-P produced similar phase-separated droplets, suggesting that phosphorylation does not significantly alter its LLPS behavior (Fig. 5F). Furthermore, the SVCV-P droplets were sensitive to the LLPS disruptor of 1,6-HEX but were not affected by 2,5-HEX (Fig. 5G). The droplets of SVCV-P labeled with AF640 also exhibited dynamic fusion activity and FRAP as detected by FRAP assay, confirming the dynamic nature of these structures and that the puncta were not artificially formed by the EGFP tag (Fig. 5H through J). Similarly, the fusion and fission, and fluorescence recovery behaviors of the SVCV-P were observed in HEK293T cells, reinforcing the LLPS characteristics of SVCV-P in a cellular context (Fig. 5K through N). To enhance *in vivo* evidence during viral infection, we further examined the inherent LLPS activity of SVCV-P in SVCV-infected EPC cells. The results confirmed that SVCV-P underwent phase separation in infected EPC cells, with phase-separated droplets similar to those observed with overexpressed SVCV-P in HEK293T cells (Fig. 6A). These findings collectively demonstrate that SVCV-P exhibits LLPS behavior both *in vitro* and *in vivo* under both experimental and viral infection conditions.

To investigate which domain contributes to the phase separation of SVCV-P, we constructed mutants lacking the IDR (SVCV-P-ΔIDR) and the central domain (CD) (SVCV-P-ΔCD) (Fig. 6B). We first observed in HEK293T cells that deletion of either the IDR or the CD regions impaired the ability of SVCV-P to form droplet puncta, with the IDR deletion having a more significant effect (Fig. 6C). We next expressed and purified the EGFP-tagged SVCV-P-ΔIDR and SVCV-P-ΔCD proteins for *in vitro* examinations. Consistent with the cellular observations, we found a significant decrease in the size of droplets from the two mutant proteins, particularly from SVCV-P-ΔIDR, compared with that of wild-type SVCV-P (Fig. 6D). This finding suggests both deletions severely disrupted phase separation, with the IDRs being the more critical regions for LLPS. Interestingly, co-incubation of SVCV-P-ΔIDR and SVCV-P-ΔCD with wild-type SVCV-P markedly restored the mutant droplet size to be comparable to that of the control wild-type SVCV-P (Fig. 6E). This suggests that wild-type SVCV-P functions as a scaffold protein in driving phase separation, and it compensates for the loss of functional domains in mutant proteins by providing additional IDR and CD regions for domain-domain interactions during phase separation. Notably, despite a significant decline in phase separation ability, the SVCV-P-ΔCD mutant still has partial droplet-forming capacity. Because the CD is necessary for the oligomerization of SVCV-P, we suggest that SVCV-P-ΔCD droplets were restricted to monomeric forms due to the loss of the CD region. Consequently, it seems reasonable to hypothesize that oligomerization may impair the fluidity of SVCV-P droplets, whereas the monomeric form benefits their fluidity. To test this hypothesis, we compared the droplet dynamics of wild-type SVCV-P and the SVCV-P-ΔCD by the FRAP assay. As expected, SVCV-P-ΔCD droplets exhibited rapid fluorescence recovery within 0.5 h or after 2 h (Fig. 6F), indicating high fluidity of the mutant protein. In contrast, wild-type SVCV-P droplets showed slower recovery, with little to no fluorescence recovery within 0.5 h or after 2 h (Fig. 6F), suggesting that these droplets adopt a more solid-like, immobile state over time. This suggests that the CD domain plays an important role in restricting the dynamics and fluidity of SVCV-P phase-separated droplets by inducing oligomerization.

### SVCV-P is an alternative scaffold protein in driving TBK1 phase separation

**Fig 7:**
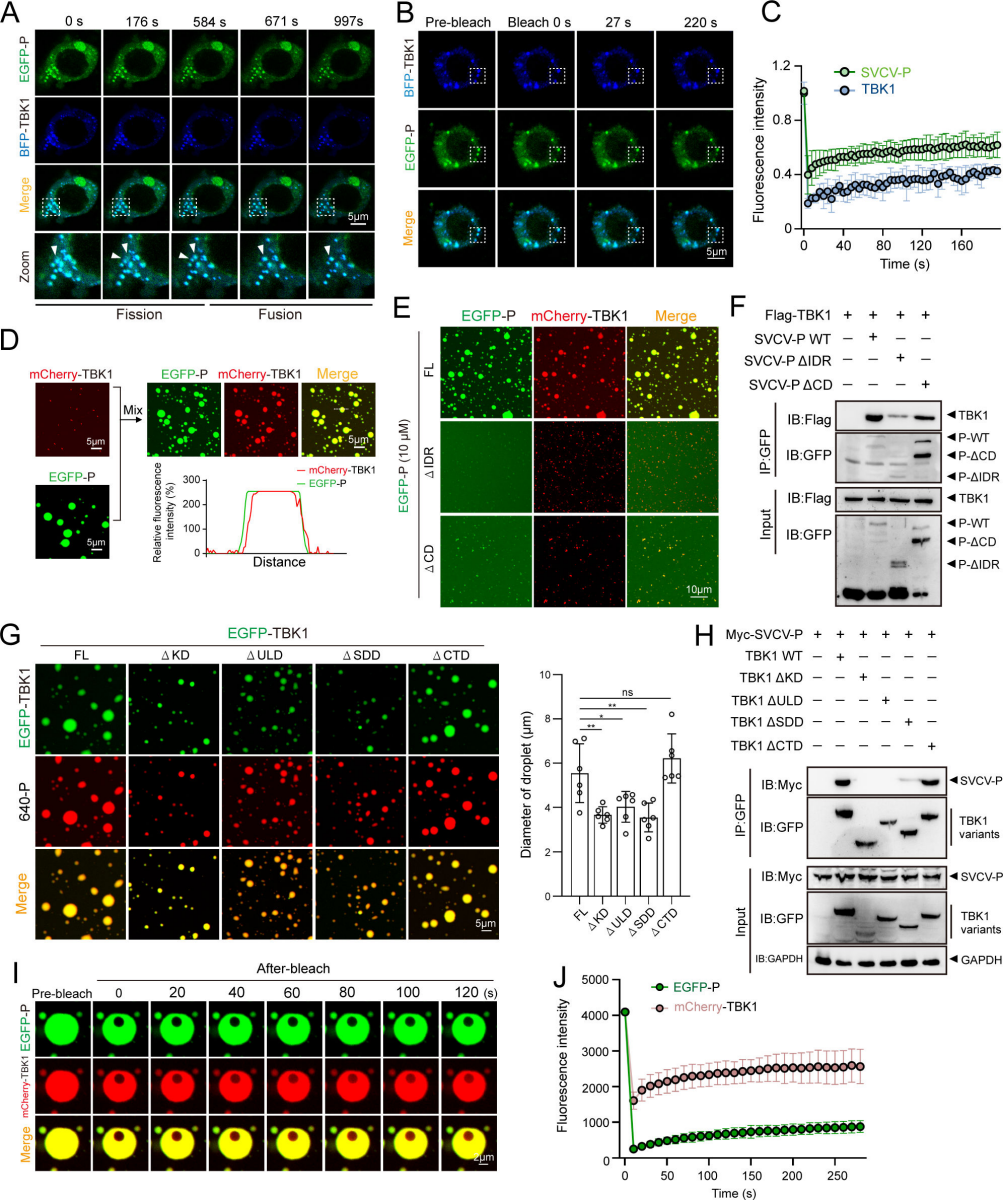
TBK1 acts as a client protein in SVCV-P-driven phase separation. (**A**) Fission (left) and fusion (right) events of co-localized condensates in HEK293T cells co-expressing EGFP-SVCV-P and BFP-TBK1. Images were acquired at the indicated time points. Scale bar: 5 µm. (**B and C**) Representative FRAP images of GFP-SVCV-P/BFP-TBK1 condensate *in vivo* (**B**) and quantification (**C**) of EGFP-SVCV-P puncta recovery. Scale bar: 5 µm. (**D**) Coalescence of preformed mCherry-TBK1 and EGFP-SVCV-P condensates into biphasic condensates *in vitro*. Fluorescence line profile shown below. (**E**) Fluorescence microscopy images of mCherry-TBK1 with EGFP-SVCV-P, EGFP-SVCV-P-ΔIDR, and EGFP-SVCV-P-ΔCD protein. Scale bar: 10 µm. (**F**) Co-IP analysis of TBK1 interaction with SVCV-P mutants. HEK293T cells were transfected with the indicated plasmids overnight, and the cell lysates were immunoprecipitated with anti-GFP antibody, followed by immunoblotting with anti-Flag antibodies. (**G**) Droplet formation analysis of co-incubated GFP-TBK1 (FL or mutants) and 640-labeled SVCV-P. Right, quantitative analysis of mixed droplet diameters (*n* = 6 per group; ^*^P < 0.05; ^**^P < 0.01; ns, not significant). Scale bar: 5 µm. (**H**) Involvement of TBK1’s N-terminal kinase and ULDs in SVCV-P interaction. HEK293T cells were transfected with the indicated plasmids for 24 h. The cell lysates were immunoprecipitated with anti-GFP antibody and immunoblotted with anti-Myc antibody. (**I and J**) Representative *in vitro* FRAP images of EGFP-SVCV-P-mCherry-TBK1 condensates (**I**) and quantification of their fluorescence recovery kinetics (**J**) (*n*  =  3 droplets). Images were captured under a confocal laser scanning microscope (Olympus FV3000, original magnification, ×630). Scale bar: 5 µm.

Given the phase-separation activity of SVCV-P and its association with TBK1, we propose that SVCV-P may act as an alternative scaffold protein that recruits the TBK1 client protein into liquid droplets by competing with IRF3, thus leading to the formation of biphasic SVCV-P-TBK1 condensates. To test this hypothesis, we co-expressed EGFP-SVCV-P and BFP-TBK1 in HEK293T cells. Confocal microscopy revealed that EGFP-SVCV-P and BFP-TBK1 dynamically formed phase-separated co-condensates, exhibiting fusion and fission activities (Fig. 7A). FRAP analysis indicated that both EGFP-SVCV-P and BFP-TBK1 within the co-localized condensates showed simultaneous recovery but at a slower rate than expected (Fig. 7B and C), suggesting that TBK1 is indeed recruited into SVCV-P condensates. We further confirmed this by co-incubating mCherry-TBK1 with EGFP-SVCV-P *in vitro*. TBK1 was observed to be recruited into SVCV-P-induced phase-separated droplets (Fig. 7D). To elucidate the molecular basis of the SVCV-P-TBK1 biphasic condensates, we investigated domain-domain interactions among the wild-type proteins and mutants lacking IDR and CD in SVCV-P, as well as KD, ULD, SDD, and CTD in TBK1. The droplet sizes in these protein combinations were assessed to determine their *in vitro* phase separation activities. Deletion of the IDR and CD regions in SVCV-P markedly impaired SVCV-P-TBK1 condensate formation (Fig. 7E), while deletion of the KD, ULD, and SDD regions in TBK1 reduced condensate size to varying degrees (Fig. 7G). These observations were corroborated by Co-IP assays (Fig. 7F and H), implicating complex intramolecular and intermolecular associations involving the IDR and CD regions of SVCV-P with the KD, ULD, and SDD regions of TBK1. The SDD region, which promotes oligomerization of TBK1, was critical for condensate formation, as its deletion reduced condensed particle size (Fig. 7G). Thus, TBK1’s intramolecular oligomerization is involved in SVCV-P-TBK1 co-phase separation. Finally, FRAP analysis revealed slow fluorescence recovery for both SVCV-P and TBK1 in the co-phase condensates (Fig. 7I and J), indicating limited mobility due to oligomerization, which may reduce fluidity beyond promoting condensation.

### SVCV-P-TBK1-IRF3 ternary coalescence

**Fig 8:**
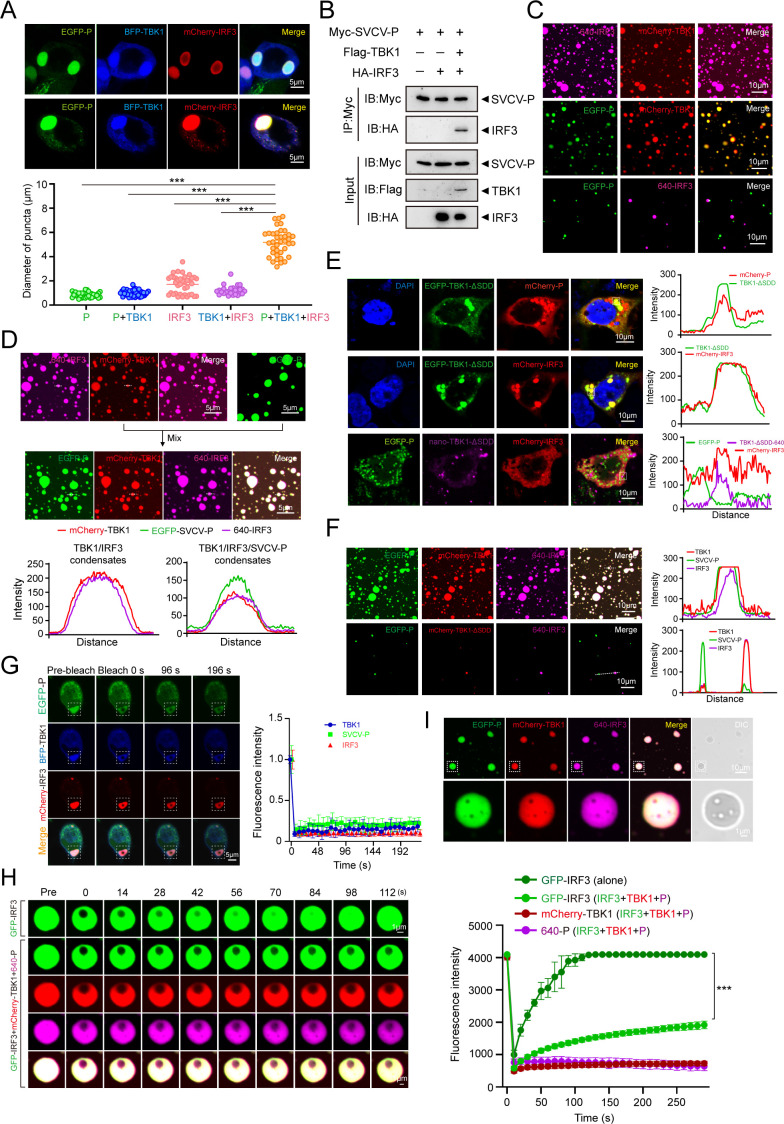
Formation of ternary SVCV-P-TBK1-IRF3 condensates *in vitro* and *in vivo*. (**A**) Confocal microscopy images showing the co-localization of EGFP-SVCV-P with BFP-TBK1 and mCherry-IRF3 (top). Quantitative analysis of puncta diameters formed by each protein alone or in combination (bottom; ^***^P < 0.001). Scale bar: 5 µm. (**B**) Co-IP analysis of competitive interactions among SVCV-P, TBK1, and IRF3. HEK293T cells were transfected with the indicated plasmids for 36 h. The cell lysates were immunoprecipitated with anti-Myc antibody and immunoblotted with anti-HA antibody. (**C**) Fluorescence image of mCherry-TBK1 with EGFP-SVCV-P or 640-IRF3, and EGFP-SVCV-P with 640-IRF3 mixed at room temperature *in vitro*. (**D**) Coalescence of preformed mCherry-TBK1 and 640-IRF3 condensates with EGFP-SVCV-P into ternary condensates *in vitro*. Line profile of fluorescence is shown at the bottom. (**E**) Confocal microscopy images of HEK293T cells co-transfected for 24 h with EGFP-TBK1-ΔSDD and mCherry-IRF3, or EGFP-TBK1-ΔSDD and mCherry-SVCV-P, or EGFP-SVCV-P with nano-TBK1-ΔSDD and mCherry-IRF3. Right, intensity profiles of the indicated proteins along the plotted lines, as analyzed by ImageJ line scan software. Images were captured under a confocal laser scanning microscope (Olympus FV3000, original magnification, ×630). Scale bar: 10 µm. (**F**) Coalescence of preformed EGFP-SVCV-P and 640-IRF3 with mCherry-TBK1 or mCherry-TBK1-∆SDD into ternary condensates *in vitro*. Scale bar: 10 µm. (**G**) The fluorescence intensity of SVCV-P-TBK1-IRF3 condensates recovered after bleaching during the FRAP assay (Left). Quantification of fluorescence intensity recovery in the bleached region of SVCV-P-TBK1-IRF3 condensates (Right). Scale bar, 5 µm. (**H**) Representative images of FRAP (left) and quantification (right) of GFP-IRF3 in the presence or absence of mCherry-TBK1 and 640-SVCV-P over a 300-s time course (*n*  =  3 droplets). Scale bar: 5 µm. (**I**) SVCV-P-TBK1-IRF3 formed heterogeneous ternary condensates *in vitro*. Images were captured under a confocal laser scanning microscope (Olympus FV3000, original magnification, ×630).

Given that both SVCV-P and IRF3 independently possess LLPS capabilities, we are prompted to explore the interplay between TBK1 phase separation events driven by SVCV-P and those triggered by IRF3 during a genuine SVCV infection. We first co-expressed EGFP-SVCV-P, BFP-TBK1, and mCherry-IRF3 in HEK293T cells to examine the co-localization of SVCV-P, TBK1, and IRF3. As expected, EGFP-SVCV-P, BFP-TBK1, and mCherry-IRF3 were clearly co-localized and formed large condensates as detected under confocal microscopy (Fig. 8A). Interestingly, IRF3 exhibited two distinct localization patterns: one where it co-distributed uniformly with TBK1 and SVCV-P; the other where IRF3 was located on the periphery of phase-separated SVCV-P-TBK1 condensates. Notably, the size of the ternary condensates formed by SVCV-P, TBK1, and IRF3 was significantly larger (5.16 ± 1.06 µm) than the biphasic condensates formed by SVCV-P and TBK1 (0.93 ± 0.26 µm) or IRF3 and TBK1 (1.21 ± 0.38 µm), and condensates formed by only SVCV-P (0.83 ± 0.19 µm) and IRF3 (1.57 ± 0.85 µm) (Fig. 8A). Moreover, the Co-IP assay showed that no interaction was detected between SVCV-P and IRF3 in HEK293T cells co-expressed with only Myc-tagged SVCV-P and HA-tagged IRF3, while the interaction between SVCV-P and IRF3 became evident upon co-expression of TBK1 (Fig. 8B). These results suggested that SVCV-P did not interact with IRF3 directly; however, it associates with IRF3 through the mediation of TBK1 in an indirect manner. Thus, TBK1 may act as a mediator that connects SVCV-P and IRF3 to promote the formation of large ternary condensates. To provide further evidence for this hypothesis, we conducted a series of *in vitro* assays to examine the co-phase separation behaviors in detail among recombinant SVCV-P, IRF3, and TBK1 proteins. We first assessed the phase separation between pairs of these proteins. Consistent with the cellular observations, mCherry-TBK1 could phase separate with either EGFP-SVCV-P or 640-IRF3, but EGFP-SVCV-P and 640-IRF3 alone could not form a phase-separated condensate (Fig. 8C). However, the addition of SVCV-P to the TBK1-IRF3 condensate resulted in the coalescence of TBK1-IRF3 and SVCV-P into a single phase (Fig. 8D). To investigate the molecular mechanism underlying the co-condensation of SVCV-P, TBK1, and IRF3, we focused on the regulatory role of SDD of TBK1 during the co-phase separation, because SDD is responsible for TBK1’s dimerization, which we hypothesize plays a critical role in organizing co-phase separation. Subcellular localization analysis showed that EGFP-TBK1-∆SDD was co-localized with either mCherry-SVCV-P or mCherry-IRF3 when they co-expressed in HEK293T cells, suggesting SDD does not affect the association between TBK1 and SVCV-P or TBK1 and IRF3 in pairs (Fig. 8E). However, minimal co-localization was observed in cells co-expressed with EGFP-TBK1-∆SDD, mCherry-SVCV-P, and mCherry-IRF3, in contrast to the immense co-localization observed in cells co-expressed with wild-type TBK1, SVCV-P, and IRF3, suggesting that the absence of SDD in TBK1 disrupted the formation of the ternary condensates (Fig. 8E). In accordance with these cellular observations, co-incubation of EGFP-tagged wild-type TBK1, mCherry-SVCV-P, and IRF3-640 formed typical ternary condensates *in vitro*; whereas minimal ternary condensates were observed when co-incubation of EGFP-TBK1-∆SDD, mCherry-SVCV-P, and IRF3-640 (Fig. 8F), reinforcing the essential role of SDD in SVCV-P-TBK1-IRF3 condensate formation. Following photobleaching, none of the three proteins in SVCV-P-TBK1-IRF3 condensates showed significant recovery (Fig. 8G), indicating their limited dynamics in the ternary state, especially IRF3, while phase-separated either alone or in combination with TBK1, now showed little to no recovery after photobleaching, indicating reduced dynamics in these phase-separated states. Additionally, FRAP analysis *in vitro* also revealed that IRF3, when co-phase separated with TBK1 and SVCV-P, exhibited significantly reduced mobility compared to when it was phase-separated alone (Fig. 8H). Notably, we observed that the ternary condensates formed by SVCV-P-TBK1-IRF3 *in vitro* were heterogeneous in structure (Fig. 8I), suggesting complex molecular organization within the condensates.

**Fig 9:**
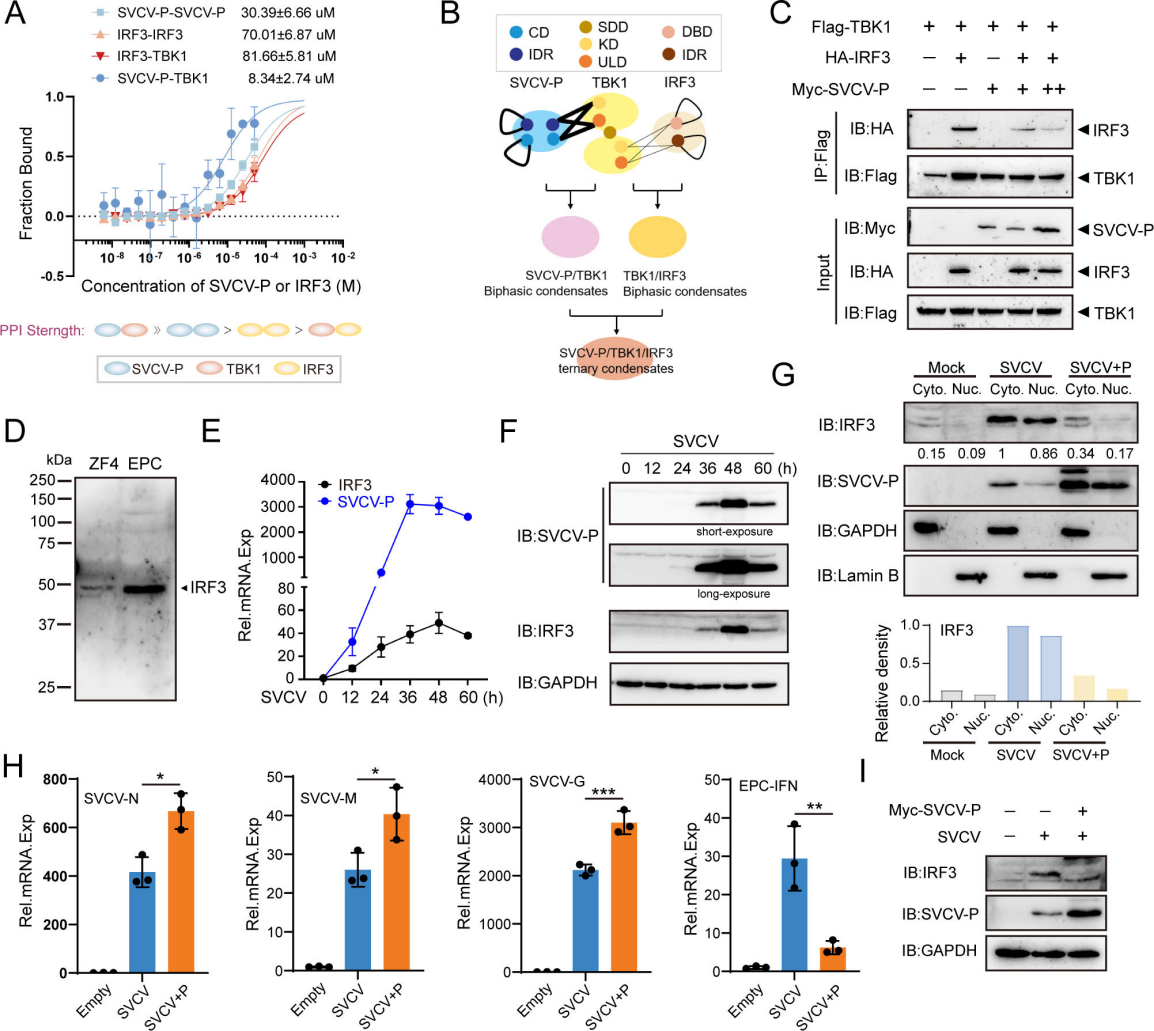
SVCV-P inhibits IRF3 nuclear translocation to suppress innate antiviral immunity. (**A**) Binding affinities between SVCV-P and TBK1, IRF3 and TBK1, SVCV-P and SVCV-P, and IRF3 and IRF3 proteins were measured by MST assay. Mean dissociation constants (*K*_d_) are provided, with data representing the mean ± SD from three independent experiments. (**B**) A proposed network model illustrating the role of relative PPI strengths in multiphase organization. Monovalent interaction sites (circles) and PPIs (lines) are shown, with thicker line weights indicating stronger interactions. Homotypic PPIs drive LLPS of SVCV-P and IRF3, while heterotypic PPIs (SVCV-P/TBK1; TBK1/IRF3) enable co-phase separation of these proteins. TBK1 enhances the multiphase coalescence of SVCV-P and IRF3. (**C**) Interaction between SVCV-P and IRF3. HEK293T cells were co-transfected with Flag-TBK1, HA-IRF3, and varying amounts of Myc-tagged SVCV-P plasmids. The cell lysates were immunoprecipitated with anti-Flag antibody and immunoblotted with anti-HA antibody. (**D**) IRF3 protein expression in SVCV-infected cells. EPC and ZF4 cells were seeded into 6-well plates overnight and infected with 100 TCID_50_ of SVCV (5 × 10^6^ TCID_50_/mL), followed by Western blotting using rabbit anti-IRF3 antibody. (**E and F**) Time-course analysis of IRF3 mRNA/protein expression during SVCV infection versus SVCV-P mRNA/protein expression. EPC cells infected with SVCV (as above) were harvested at indicated times (0–60 h) for RT-qPCR or immunoblotting. (**G**) Localization of IRF3 and SVCV-P in SVCV-infected EPC cells. Cytoplasmic (Cyto.) and nuclear (Nuc.) fractions from mock-treated, SVCV-infected, or SVCV-P-overexpressing EPC cells were immunoblotted for IRF3, SVCV-P, GAPDH, and Lamin B. Blots were re-probed using ImageJ for GAPDH and Lamin B as loading controls. (**H**) mRNA levels of SVCV-N, SVCV-M, SVCV-G, and EPC-IFN genes in samples from (**G**), analyzed by RT-qPCR. Data represent mean ± SD from three independent experiments (^*^P < 0.05; ^**^P < 0.01; ^***^P < 0.001). (**I**) The IRF3 protein levels decreased in SVCV-infected or SVCV-P-overexpressing EPC cells. The expression of IRF3 and SVCV-P in cells infected with SVCV and transfected with a plasmid expressing pCMV-Myc-SVCV-P was analyzed using Western blotting, as described above.

To investigate the molecular basis of the observed heterogeneity in SVCV-P-TBK1-IRF3 condensates, we performed Attenuated Total Reflection Fourier-Transform Infrared Spectroscopy (ATR-FTIR) analysis, focusing on the amide I region (1,600–1,700 cm⁻¹) of the phase-separated samples at different incubation times. Both spectra (Fig. S1) displayed a prominent amide I band between 1,600 and 1,700 cm⁻¹. Compared with the freshly formed (0 h) condensates, the spectrum after 24 h of incubation exhibited a clear shift of the amide I maximum from approximately 1,645 cm⁻¹ to lower wavenumbers (around 1,630 cm⁻¹), accompanied by an increase in absorbance at the β-sheet region. This shift toward lower frequencies indicates enhanced intermolecular hydrogen bonding and the accumulation of β-sheet-rich structures, a conformational change associated with stable cross-linking and irreversible protein aggregation, suggesting that the condensates progressively evolve from a liquid-like to a more ordered, solid-like state over time. These results suggest that SVCV-P, TBK1, and IRF3 can co-phase separate into a single condensate with increased solid-like properties, within which IRF3 loses much of its mobility. To further explore the potential molecular underpinnings of the formation of ternary condensates and their solidity-like phase transition, we quantified the equilibrium dissociation constants (*K*_d_) of protein-protein interactions (PPIs) among SVCV-P, TBK1, and IRF3 proteins using MST. The results indicated that heterotypic SVCV-P-TBK1 interactions (*K*_d_ = 8.34 ± 2.74 µM) were significantly stronger than those between TBK1 and IRF3 (*K*_d_ = 81.66 ± 5.81 µM). Furthermore, under identical conditions, homotypic SVCV-P-SVCV-P interactions (*K*_d_ = 30.39 ± 6.66 µM) were more robust than IRF3-IRF3 interactions (*K*_d_ = 70.01 ± 6.87 µM) (Fig. 9A). This suggests that during the infection process of SVCV, SVCV-P has a high affinity for TBK1. Given the binding strength hierarchy, SVCV-P-TBK1 >SVCV-P-SVCV-P >IRF3-IRF3 > IRF3-TBK1, we propose that SVCV-P preferentially associates with TBK1 and subsequently recruits IRF3-TBK1 droplets to organize the ternary coalescence of SVCV-P-TBK1-IRF3 (Fig. 9B). To support this hypothesis, competitive Co-IP assays revealed that SVCV-P partially inhibited the interaction between TBK1 and IRF3 (Fig. 9C).

### SVCV-P-TBK1-IRF3 coalescence suppresses the IFN-inducing pathway

Given the critical roles of TBK1 and IRF3 in RIG-I/MAVS-initiated IFN-inducing signaling pathway, we explored the role of SVCV-P-TBK1-IRF3 co-phase separated condensates during SVCV infection. Using a time-course approach following SVCV infection, we analyzed the dynamics of SVCV-P and endogenous IRF3 expression at six time points using RT-qPCR and Western blot by utilizing polyclonal antibodies prepared in our study (Fig. 9D). We observed that the expression pattern of IRF3 closely mirrored that of SVCV-P (Fig. 9E and F). Given that the dimerization of IRF3 upon phosphorylation by TBK1 and subsequent nuclear translocation is crucial for IFN gene expression through the IFN-inducing signaling pathway, we investigated the regulatory role of SVCV-P in IRF3 nuclear translocation under conditions of SVCV infection and overexpression of SVCV-P in EPC cells. Through nucleus and cytoplasmic fractionation, we detected changes in the ratio of IRF3 in the nucleus to that in the cytoplasm. The results revealed that both viral infection and overexpression of SVCV-P significantly decreased IRF3 levels in the nucleus, indicating that SVCV-P inhibits IRF3 nuclear translocation (Fig. 9G). These findings suggest that SVCV-P suppresses the IFN-inducing signaling pathway by preventing nuclear import of IRF3, thereby downregulating IFN expression. In support of this notion, inhibition of IRF3 by SVCV-P significantly reduced the IFN expression level, which was accompanied by the increased expression levels of the viral *n*, *m*, and *g* genes of SVCV (Fig. 9H). Additionally, we observed a reduction in total IRF3 protein levels in EPC cells overexpressing SVCV-P (Fig. 9I). This implies that SVCV-P, along with its associated TBK1 and IRF3 complexes, might be targeted for autophagic degradation during viral infection, as aggregated proteins are frequently cleared by this pathway (39–41). However, this potential mechanism remains speculative and highlights an intriguing avenue for future research. Collectively, our results indicate that the SVCV-P protein disrupts the IRF3-mediated immune response by inhibiting IRF3 nuclear translocation and reducing the expression of the IFN gene in SVCV infection.

### Role of phase separation in SVCV-P-suppressed TBK1-IRF3 activation

**Fig 10:**
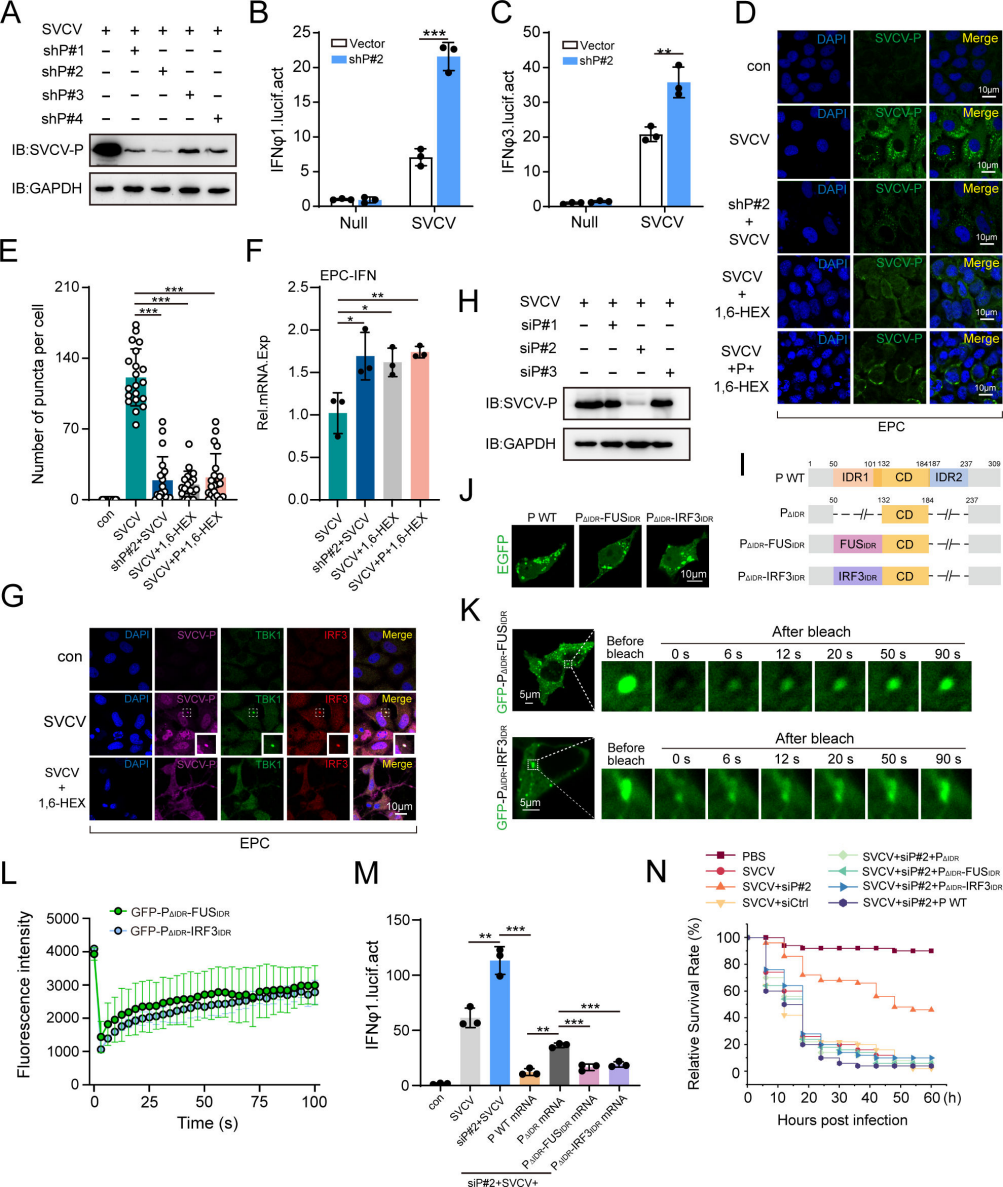
Regulation of SVCV-P in IFN responses via phase separation *in vivo*. (**A**) Knockdown efficiency of shRNAs targeting different sequences of the SVCV-P mRNA. EPC cells transfected with shRNAs against SVCV-P mRNA (shP#1-#4) were infected with 100 TCID_50_ of SVCV (5 × 10^6^ TCID_50_/mL) at 12 h post-transfection. SVCV-P expression was assessed by immunoblotting with an anti-SVCV-P antibody. (**B, C**) Effect of SVCV-P knockdown on IFN promoter activation. EPC cells were transfected with shP#2 (500 ng), IFN-φ1 or IFN-φ3 promoter reporter (250 ng), and pRL-TK (25 ng). Following a 24-h incubation, cells were infected with SVCV (100 TCID₅₀). Luciferase activity was measured 24 h post-infection. (**D**) Examination of SVCV-P droplet formation in SVCV-infected cells. EPC cells transfected with shP#2 or Myc-SVCV-P-encoding plasmid were infected with SVCV (100 TCID₅₀) for 24 h, treated with 1,6-HEX, and immunostained with an anti-SVCV-P antibody. Images were acquired using confocal microscopy (Olympus FV3000; ×630 magnification). Scale bar: 10 µm. (**E**) The number of SVCV-P puncta per cell was quantified in randomly selected fields from the images in (**D**). (**F**) Total RNA extracted from cells in (**D**) was analyzed to determine the expression levels of the IFN gene in EPC cells. (**G**) Colocalization of SVCV-P, TBK1, and IRF3 was visualized using confocal microscopy. EPC cells infected with SVCV (100 TCID_50_) and treated with or without 1,6-HEX were immunostained with anti-SVCV-P, anti-TBK1, and anti-IRF3 antibodies. Images were captured as described in (**D**). Scale bar: 10 µm. (**H**) Knockdown efficiency of siRNAs targeting different sequences of the SVCV-P mRNA. EPC cells transfected with siRNAs (100 nM) against SVCV-P mRNA (siP#1-#4) were infected with SVCV (100 TCID_50_) at 12 h post-transfection. SVCV-P expression was assessed by immunoblotting with an anti-SVCV-P antibody. (**I**) Schematic of SVCV-P protein variants. (**J**) Subcellular localization of EGFP-SVCV-P, EGFP-SVCV-P_ΔIDR_-FUS_IDR_, and EGFP-SVCV-P_ΔIDR_-IRF3_IDR_ in HEK293T cells. Scale bar: 10 µm. (**K and L**) FRAP analysis of EGFP-SVCV-P_ΔIDR_-FUS_IDR_ and EGFP-SVCV-P_ΔIDR_-IRF3_IDR_ puncta *in vivo*. Representative recovery images (**K**) and quantification (**L**). Scale bar: 5 µm. (**M**) IFNφ1 promoter activity in zebrafish embryos. Embryos were microinjected with siP#2 (100 nM), indicated mRNAs (150 pg/embryo), IFNφ1-pro (100 pg/embryo), and pRL-TK (10 pg/embryo) reporter vectors. At 12 h post-injection, embryos were untreated or treated with SVCV (2 nL/embryo; 5 × 10^6^ TCID_50_/mL). Luciferase activities were monitored 24 h after infection. (**N**) Survival analysis of zebrafish embryos. Embryos were microinjected with siP#2 and indicated mRNAs prior to infection with SVCV as described above. Statistical differences were analyzed using the log-rank test (*n* = 50). Data are representative of three independent experiments and are shown as means ± SD (^*^P < 0.05; ^**^P < 0.01; ^***^P < 0.001; NS, not significant).

To confirm the regulatory role of SVCV-P-mediated phase separation in impairing TBK1/IRF3 activation within the IFN-inducing signaling cascade during SVCV infection, a knockdown assay was initially conducted using four distinct shRNAs (labeled as shP#1, shP#2, shP#3, shP#4), each targeting unique regions of the mRNA transcripts originating from the SVCV-P encoding gene. Western blot analysis revealed that these shRNAs collectively diminished SVCV-P expression to differing extents, with shP#2 yielding the most significant effect (Fig. 10A). Consequently, shP#2 was selected for subsequent knockdown experiments. Upon SVCV infection in EPC cells, the knockdown of SVCV-P using shP#2 rescued the activity of IFNφ1-pro and IFNφ3-pro reporters (Fig. 10B and C), indicating restoration of IFN-inducing signaling. IF imaging assay further demonstrated that phase separation droplets induced by SVCV-P during SVCV infection were significantly reduced following shP#2-mediated knockdown of SVCV-P (Fig. 10D and E). Additionally, treatment with 1,6-HEX also significantly attenuated the formation of these droplets (Fig. 10D and E). Notably, in 1,6-HEX-treated SVCV-infected cells, overexpression of SVCV-P failed to reconstitute visible droplet formation, indicating that SVCV-P-induced LLPS remains suppressed under sustained inhibitory conditions, even with abundant exogenous SVCV-P expression (Fig. 10D and E). Correspondingly, IFN expression was significantly upregulated, reinforcing the idea that inhibition of IFN-inducing signaling mediated by SVCV-P phase separation was partially relieved (Fig. 10F). To further investigate the interaction between SVCV-P, TBK1, and IRF3 during SVCV infection, we performed IF experiments using antibodies specific to SVCV-P, IRF3, and TBK1 proteins. As anticipated, we observed the formation of SVCV-P-TBK1-IRF3 ternary condensates during SVCV infection, and these condensates were reduced by 1,6-HEX treatment (Fig. 10G), further supporting the role of phase separation in the IFN-inducing pathway.

Finally, we conducted *in vivo* assays to validate the repressive role of SVCV-P-mediated phase separation in IFN induction during SVCV infection using zebrafish embryos as a model organism. For this purpose, zebrafish embryos were challenged with 100 TCID_50_ SVCV (TCID_50_ = 5 × 10^6^ TCID_50_/mL), and the transcripts of the viral *p* gene were silenced via small interfering RNAs (siRNA). Three SVCV-P-targeting siRNA (labeled as siP#1, siP#2, and siP#3) were designed, and their knockdown efficacy was evaluated in SVCV-infected EPC cells. Western blot analysis revealed that siP#2 exhibited the most potent silencing effect (Fig. 10H) and thus was chosen for the subsequent assessments. For functional rescue assays, we generated mRNAs encoding wild type (P_WT_) and two chimeric SVCV-P proteins (P_△IDR_-FUS_IDR_ and P_△IDR_-IRF3_IDR_), wherein the latter’s two IDRs of SVCV-P were replaced with the IDR from FUS (a π-π stacking-driven phase separation protein) or from IRF3 (an electrostatic interaction-driven phase separation protein), respectively (Fig. 10I). When expressed in HEK293T cells, both chimeric proteins demonstrated LLPS activities comparable to the wild-type protein (Fig. 10J–L), reinforcing that SVCV-P undergoes IDR-driven LLPS through mechanisms analogous to classical phase-separating proteins. In rescue experiments, SVCV-P knockdown embryos (achieved by siP#2 injection) were administered mRNAs encoding P_WT_, P_△IDR_, P_△IDR_-FUS_IDR_, or P_△IDR_-IRF3_IDR_ prior to SVCV challenge. Luciferase reporter assays revealed that the SVCV-P knockdown significantly enhanced IFNφ1-pro reporter activity (Fig. 10M), which correlated with a dramatically increased zebrafish embryos’ survival rate (Fig. 10N). However, this IFN activation was substantially attenuated by reintroduction of P_WT_, P_△IDR_-FUS_IDR_, or P_△IDR_-IRF3_IDR_, with comparable suppression levels across rescue groups (Fig. 10M). Notably, P_△IDR_ alone lost a significant portion of its IFN inhibitory function compared to the chimeric variants P_△IDR_-FUS_IDR_ and P_△IDR_-IRF3_IDR_, establishing the essential role of IDR-mediated phase separation in SVCV-P function (Fig. 10M). These observations were further corroborated by the survival rate analyses of SVCV-infected embryos (Fig. 10N). Collectively, our results provide compelling *in vivo* evidence supporting the inhibitory function of SVCV-P in the IFN-inducing signaling pathway through an LLPS-dependent mechanism.

## DISCUSSION

LLPS is a fundamental cellular physiological process that regulates numerous biological activities (32). Dysfunctions in LLPS have been implicated in various diseases, including neurodegenerative disorders and cancer (42, 43). Recent studies have highlighted the critical role of LLPS in viral replication and immune evasion. For instance, the coronavirus hijacks FAM134B and ATL3 into p62 condensates to facilitate viral replication (44). Adenoviruses employ LLPS to create replication compartments, which shield viral RNA from host immune detection while boosting genome replication (35). Similarly, proteins such as RABV N, HIV Gag, and measles virus N/P interact with viral RNA to form phase-separated condensates for genome packaging and assembly (45–50). Influenza A virus utilizes Rab11 to form condensates, thereby enhancing the kinetics of genome assembly (51). Additionally, herpesvirus ORF52 and VP22/ORF9 proteins disrupt cGAS-DNA phase separation to prevent activation of cGAS-mediated antiviral immune responses and facilitate viral immune evasion (52, 53). Furthermore, the NSs protein of the thrombocytopenia syndrome virus sequesters IRF7 into viral inclusion bodies and isolates TBK1 and IKKε from MAVS, suppressing the induction of IFN-α and IFN-β, which helps the virus evade the host immune response (54). In this study, we elucidate a novel mechanism whereby TBK1 and IRF3 form droplet condensates via LLPS, which enhances kinase-substrate proximity, thereby facilitating IRF3 phosphorylation and promoting the expression of IFN genes. Conversely, the SVCV-P inhibits IFN production by disrupting TBK1-IRF3 phase separation. These findings provide new insights into a precise docking mechanism mediated by LLPS between TBK1 and IRF3, and they demonstrate a novel strategy for SVCV’s immune evasion by targeting this crucial hub in the RIG-I/MAVS-triggered IFN-inducing pathway.

To elucidate the mechanism underlying phase separation between IRF3 and TBK1, we examined the role of wild-type IRF3 and TBK1 as well as individual domains in this process by generating a series of mutants devoid of specific functional domains. The results indicated that IRF3 exhibits significant phase-separation activity both *in vitro* and *in vivo*, with the IDR and DBD playing crucial roles. This suggests that IRF3 acts as a scaffold protein driving IRF3-TBK1 phase separation. In contrast, TBK1 lacks autonomous phase-separation capacity. However, when paired with IRF3, co-phase separation of the two proteins was observed. This confirms that TBK1 functions as a client protein, recruited into phase-separated condensates facilitated by the IRF3 scaffold protein. Notably, co-phase separation activity was abolished when IDR or DBD-deficient IRF3 mutants were combined with TBK1 mutants lacking the KD, ULD, or SDD. These findings indicate that the DBD and IDR of IRF3, and the KD, ULD, and SDD of TBK1 are essential components, either through direct interaction or indirect support, for IRF3-TBK1 co-phase separation. AlphaFold3 analysis corroborated that TBK1 dimerizes via SDD, with KD and ULD symmetrically distributed at both ends of the axis formed by SDD (Fig. S2A). This aligns with previous reports that SDD is involved in TBK1 dimer formation (55), while ULD serves as a linker domain facilitating spatial coordination between KD and SDD, as well as interactions with other proteins (56). Given that SDD deletion significantly impaired IRF3-TBK1 phase separation, TBK1 dimerization appears critical for this process. Next, domain interaction analysis indicated that IRF3 primarily interacts with TBK1’s KD via its DBD and IDR (Fig. 3J and K). The main forces originate from the amino acids at positions R43 and K95 of DBD and D141 and D143 of IDR, which interact with the amino acids at positions K61, E165, S93, and K137 of KD via hydrogen bonds (Fig. S2D), respectively.

The SVCV-P primarily consists of the first IDR1, CD, the second IDR2, and the C-terminal domain (CTD). Due to its dual IDRs, SVCV-P is considered a potential phase-separating protein. We proposed that it could function as an alternative scaffold to IRF3, competitively driving TBK1 co-phase separation and thereby spatially sequestering TBK1 away from IRF3, ultimately inhibiting TBK1-mediated phosphorylation of IRF3. Indeed, SVCV-P exhibited strong phase separation activity both *in vitro* and *in vivo*, with the IDRs being critical and the CD playing a partial role in this process. When combined with TBK1, SVCV-P and TBK1 underwent co-phase separation. These findings confirm that SVCV-P acts as a scaffold protein, driving TBK1 into phase-separated co-condensates. However, co-phase separation activity was impaired to varying degrees when IDR- or CD-deficient SVCV-P mutants were combined with TBK1 mutants lacking the KD, ULD, or SDD (Fig. 7E and G). This suggests that multiple interactions between the IDRs and CD of SVCV-P and the KD, ULD, and SDD of TBK1 are involved in SVCV-P-TBK1 phase separation. Given that the CD facilitates SVCV-P dimerization and the SDD mediates TBK1 dimerization, it is proposed that dimerization of both proteins contributes to the formation of SVCV-P-TBK1 condensates. AlphaFold3 analysis revealed that the CD and IDR2 of SVCV-P are positioned near the KD of TBK1, with a specific region of IDR2 interacting directly with the KD (Fig. S2E). The primary interaction forces stem from hydrogen bonds between the C224 and R226 of IDR2 and the R308 and E301 of KD (Fig. S2E).

Since both IRF3 and SVCV-P drive TBK1 phase separation, with IRF3 facilitating IFN production and SVCV-P inhibiting it by disrupting IRF3-TBK1 phase separation, we hypothesized that during SVCV infection, IRF3-TBK1 and SVCV-P-TBK1 droplets might interact and remodel into a new phase-separated state. To test this, we combined recombinant SVCV-P, TBK1, and IRF3 proteins *in vitro*, observing the formation of distinct ternary co-condensates. Similarly, co-expression in HEK293T or EPC cells also yielded clear SVCV-P-TBK1-IRF3 co-condensates. These ternary complexes were significantly larger than their binary counterparts (IRF3-TBK1 or SVCV-P-TBK1) and exhibited slowed or nearly abolished FRAP dynamics, suggesting a transition from LLPS to a solid-like aggregated state (LSPT). Further analysis revealed that SVCV-P and IRF3 cannot directly form co-phase-separated droplets but require TBK1 as an adaptor to form ternary condensates. When using a TBK1 mutant lacking the SDD, condensate formation was severely impaired both *in vitro* and *in vivo*, confirming that SDD bridges SVCV-P and IRF3. These findings imply that TBK1 likely dimerizes within the ternary complex, with its SDD-mediated oligomerization playing a crucial role in driving the phase transition. Additionally, while IRF3 remained highly dynamic in IRF3-TBK1 droplets, its mobility was nearly abolished in ternary condensates, indicating that SVCV-P restricts IRF3’s activity and promotes LSPT. The transition from LLPS to LSPT involves multifactorial regulation. LLPS relies on multivalent weak interactions (e.g., hydrophobic interactions and π-π stacking), whereas LSPT arises from protein conformational changes (e.g., β-sheet formation and domain exposure), enabling stable cross-linking and irreversible aggregation (57). Consistent with this notion, our ATR-FTIR analysis showed enhanced intermolecular hydrogen bonding and accumulation of β-sheet-rich structures in the solid-like SVCV-P-TBK1-IRF3 condensates. Complementary to these findings, AlphaFold3 prediction provided additional support by indicating altered protein interfaces within the ternary complex. Specifically, SVCV-P’s IDR1 and TBK1’s SDD extended parallel to TBK1’s top, while SVCV-P’s CD partially overlapped with IRF3’s IAD and ID, competitively binding TBK1’s KD region. The exposed IDRs of IRF3 and SVCV-P formed an extended interaction network, facilitating solid-state aggregation (Fig. S2B, C, and F). Additionally, the dynamic fission and fusion of IRF3-TBK1 droplets in infected cells facilitate encounters with SVCV-P-TBK1 condensates. Given the stronger binding affinity of SVCV-P-TBK1 (*K*_d_ = 8.34 ± 2.74 µM) compared to IRF3-TBK1 (*K*_d_ = 81.66 ± 5.81 µM), SVCV-P likely competes for TBK1, thereby nucleating ternary condensates. Within contact zones, SDD-mediated TBK1 oligomerization may promote droplet fusion, incorporating IRF3 into SVCV-P-TBK1 condensates and ultimately forming solidified aggregates. Two distinct IRF3 distribution patterns (i.e., concentrated at the condensate periphery or uniformly distributed throughout) support this model (Fig. 8A). The peripheral localization likely reflects initial IRF3 incorporation into the SVCV-P-TBK1 phase, whereas uniform distribution suggests advanced structural remodeling and phase fusion. Together, these findings suggest a progressive maturation process underlying ternary condensate formation and LSPT.

**Fig 11:**
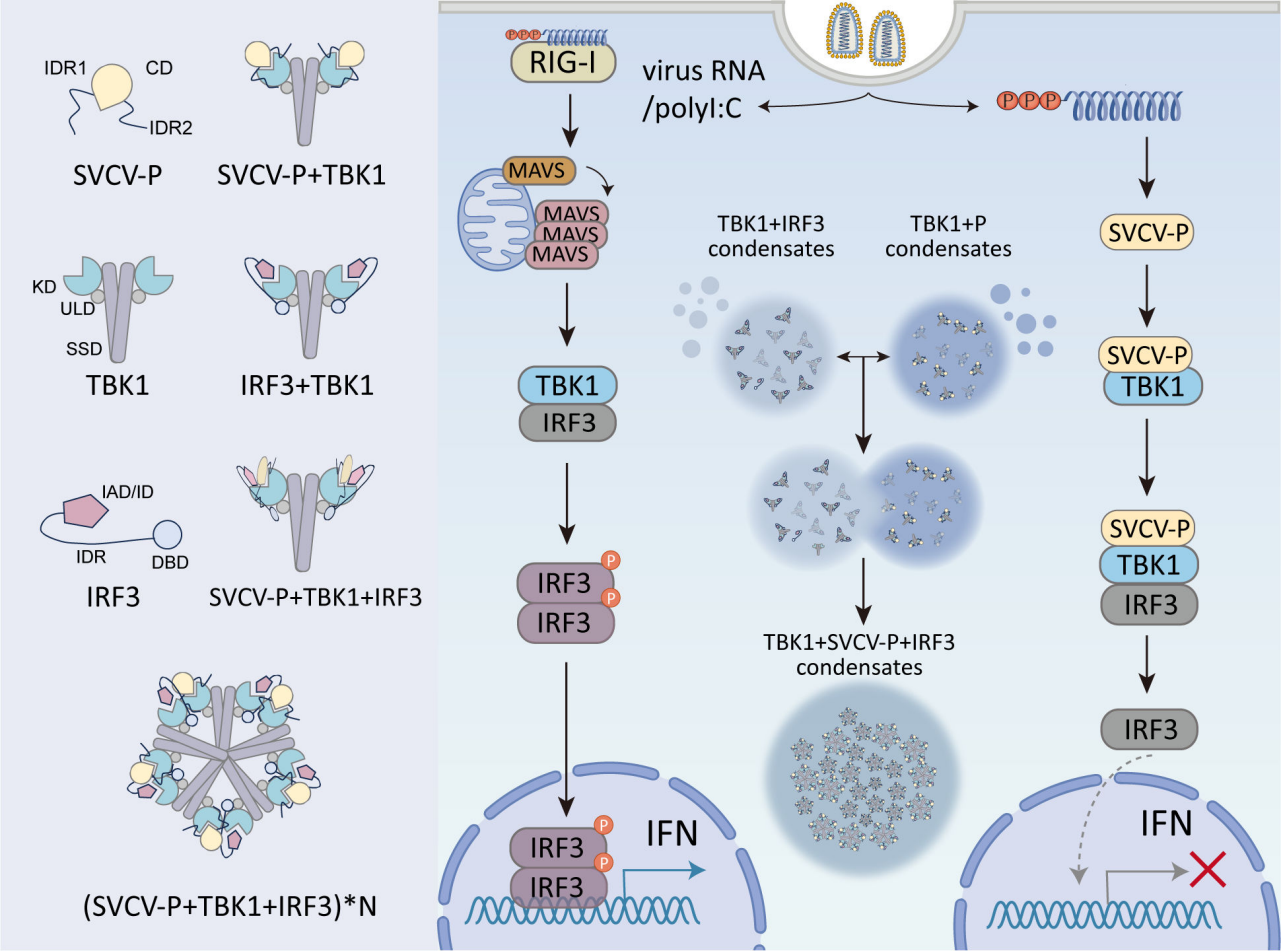
Proposed model of SVCV-P impairing antiviral IFN signaling by sequestering TBK1-IRF3 complexes via phase separation. This schematic illustrates how SVCV-P inhibits host IFN responses through LLPS. The left panel depicts simplified molecular diagrams of SVCV-P, IRF3, and TBK1, highlighting their multivalent interactions that drive higher-order complex formation. The right panel shows that upon viral RNA stimulation, the RIG-I/MAVS signaling axis activates downstream TBK1, which phosphorylates IRF3 and facilitates its nuclear translocation, ultimately driving IFN gene expression (left pathway). During this process, TBK1 and IRF3 form functional condensates through LLPS (middle pathway), a crucial step for IFN induction. SVCV-P hijacks this process by competitively sequestering TBK1 (right pathway). The dynamic activity between IRF3-TBK1 and SVCV-P-TBK1 condensates facilitates their merger into ternary IRF3-TBK1-SVCV-P aggregates (middle pathway). These pathological aggregates immobilize IRF3, thereby suppressing antiviral IFN responses.

Collectively, this study reveals that TBK1 and IRF3 form functional condensates through LLPS, thereby enhancing IRF3 phosphorylation and IFN production. SVCV-P hijacks this pathway by sequestering TBK1, forming aberrant pathological aggregates that immobilize IRF3 and suppress IFN responses (Fig. 11). Our findings uncover an LLPS-mediated docking mechanism for TBK1-IRF3 signaling and identify a viral immune evasion strategy via targeted disruption of this hub. This viral exploitation of LLPS to generate dysfunctional aggregates marks a conceptual advance in host-pathogen interactions. Although our study focuses on SVCV, the proposed mechanism may extend to other viral systems and warrants further exploration. Beyond its mechanistic implications, this work highlights potential therapeutic interventions to restore IFN defenses. Future research should define cellular regulators that govern phase transitions and explore pharmacological agents to restore physiological condensates and antiviral immunity.

## MATERIALS AND METHODS

### Experimental fish and embryos

Wild-type AB zebrafish (*Danio rerio*) with body weights of 0.5–1.0 g and lengths of 3–4 cm were maintained in recirculating water at 28°C under standard laboratory conditions as previously described (58). Zebrafish embryos and the stages of embryonic development were prepared and determined according to previous protocols (59).

### Cells and virus

Human embryonic kidney 293T (HEK293T) cells were cultured in Dulbecco’s modified Eagle’s medium (DMEM) (Corning) supplemented with 10% (vol/vol) fetal bovine serum (FBS) (Gibco, Life Technologies) at 37°C in 5% CO_2_. Spodoptera frugiperda (Sf9) cells were cultured in Sf-900 II serum-free medium (Gibco, Life Technologies) at 28°C without CO_2_. Zebrafish ZF4 cells were cultured in DMEM-F-12 cell culture medium (HyClone) with 10% (vol/vol) FBS (Bovogen Biologicals) at 28°C in 5% CO_2_. Epithelioma papulosum cyprini (EPC) cells were cultured in medium 199 (Invitrogen) supplemented with 10% (vol/vol) FBS (HyClone) at 28°C in 5% CO_2_. SVCV was propagated in EPC cells at 28°C and titrated in 96-well plates until the cytopathic effect (CPE) was complete (26), and the culture medium was collected and stored at −80°C until use. Viral titers were determined by a 50% tissue culture infective dose (TCID_50_) assay on EPC cells using the Reed-Müench method (60) and diluted to the appropriate concentration in phosphate-buffered saline (PBS), followed by the previously described protocol (26).

### Plasmid construction and reagents

The full-length SVCV-P coding sequence (CDS) was inserted into pcDNA6-Myc (Invitrogen) and pEGFP-C1 (Clontech) to construct eukaryotic expression vectors. The sequences encoding the four SVCV-P mutant proteins (named SVCV-P∆IDR, SVCV-P∆CD, SVCV-P_ΔIDR_-FUS_IDR_, and SVCV-P-P_ΔIDR_-IRF3_IDR_) were cloned into pEGFP-C1. Similarly, the CDSs of zebrafish TBK1 and IRF3 were cloned into the pCMV-HA (Invitrogen), pCMV-Flag (Invitrogen), pmCherry-N1 (Clontech), and pCMV-BFP (Clontech) or pEGFP-C1 (Clontech) vector. The CDSs of TBK1 with deletions of the N-terminal KD, ubiquitin-like domain (ULD), α-helical SDD, and CTD were constructed in pEGFP-C1 and were named pEGFP-TBK1-∆KD, pEGFP-TBK1-∆ULD, pEGFP-TBK1-∆SDD, and pEGFP-TBK1-∆CTD. Additionally, the CDS of SVCV-P, SVCV-P∆IDR, SVCV-P∆CD, and TBK1 were also constructed in pCold-EGFP-GST and pFastBac1-C-EGFP (Beyotime) for recombinant protein preparation, while SVCV-P and IRF3 were constructed in pET28a-His. The short hairpin RNA of SVCV-P was designed by BLOCK-iT RNAi Designer and cloned into the pLKO.1-Puro vector. The plasmids containing IFNφ1-Luc, IFNφ3-Luc in the pGL3-Basic luciferase reporter vector (Promega) were constructed as described previously (61). The Renilla luciferase internal control vector (pRL-TK) was purchased from Promega. The primers used for construct generation are shown in S1 Table. Plasmids for transfection and microinjection were prepared free of endotoxin by using EndoFree plasmid minikit II (Omega). For hexanediol treatment, cells were washed with PBS twice and incubated with 1% 1,6-hexanediol (catalog number 240017, Sigma) for 30 s; SVCV-P protein was treated with 5% 1,6-hexanediol or 2,5-hexanediol (catalog number H11904, Sigma) *in vitro* for 30 s. Poly(I:C) (catalog number tlrl-picwlv, Invivogen) was used at final concentrations of 1 µg/mL. Rabbit anti-TBK1 antibody (Ab) (catalog number bs-7497R; Bioss) was used at a 1:500 or 1:200 dilution for IP or IF, respectively.

### RNA extraction and real-time quantitative PCR

Total RNA was extracted using the TRIzol reagent (Invitrogen). The transcripts of the IFNφ1, IFNφ3 in embryos, and N/G/P genes of SVCV in embryos or EPC cells were analyzed via real-time quantitative PCR (RT-qPCR) on a CFX96 real-time system (Bio-Rad). In brief, all PCR experiments were performed in a total volume of 10 µL by using a SYBR green PCR master mix (TaKaRa). PCR conditions were as follows: 95°C for 2 min and then 40 cycles of 95°C for 15 s, 60°C for 15 s, and 72°C for 20 s. The relative fold changes were calculated by comparison with the corresponding controls using the 2^−∆∆Ct^ method. Each PCR trial was repeated three times. The primers used are listed in Table S1.

### Western blot analysis

HEK293T or EPC cells were treated with Western blot and IP lysis buffer containing a protease inhibitor mixture (Beyotime). The proteins were separated by 12% SDS-PAGE and transferred onto polyvinylidene difluoride transfer membranes (Millipore Sigma). The blots were blocked for 1 h at room temperature with 5% skimmed milk (Sangon), followed by incubation with mouse or rabbit anti-Flag/Myc/HA mAbs (Abcam) overnight at 4°C. After the addition of horseradish peroxidase-conjugated goat anti-rabbit/mouse IgG Ab (Abcam) for 1 h at the temperature, the immunoreactive proteins were visualized with ECL reagents (Thermo Fisher) by using a digital gel image analysis system (Tanon). The membranes were reprobed after incubation in Restore Western Blot stripping buffer (Thermo Fisher).

### Co-IP assay

The HEK293T or EPC cells seeded in 10 cm^2^ dishes overnight were transfected with a total of 6 µg of the plasmids as indicated (pCMV-TBK1-flag and pCMV-IRF3-HA; pCMV-SVCV-P-Myc and pCMV-TBK1-HA; pCMV-SVCV-P-Myc and pCMV-IRF3-Flag; pCMV-TBK1-flag and pEGFP-SVCV-P FL or pEGFP-SVCV-P ∆IDR or pEGFP-SVCV-P ∆CD; pCMV- SVCV-P-Myc and pEGFP-TBK1 FL or pEGFP-TBK1 ∆KD or pEGFP-TBK1 ∆ULD or pEGFP-TBK1 ∆SDD or pEGFP-TBK1 ∆CTD; pCMV-TBK1-flag, pCMV-IRF3-HA and pCMV-SVCV-P-Myc). At 48 h post-transfection, the medium was removed carefully, and the cells were lysed with Western blot and IP lysis buffer containing a protease inhibitor mixture (Beyotime) at 4°C for 1 h. The cellular debris was removed by centrifugation at 10,000 × *g* for 10 min at 4℃, and the supernatant was incubated with tag antibody or unrelated IgG overnight at 4°C with constant agitation. The next day, the mixtures of cell lysates and antibodies were incubated with 50 µL of protein A-agarose beads (Bio-Rad) for 4 h. The beads were washed three times with lysis buffer, and the obtained samples were further analyzed by immunoblotting with the indicated Abs.

### Preparation of recombinant proteins

A positive colony of *E. coli* BL21 (DE3) harboring pCold-EGFP-GST-SVCV-P/SVCV-P∆IDR/SVCV-P∆CD, pET28a-EGFP-SVCV-P-His, pET28a-SVCV-P/IRF3-His, pET28a-EGFP-IRF3/∆DBD/∆IDR/∆IAD/∆ID-His, and pET28a-EGFP-TBK1//∆KD /∆ULD/∆SDD/∆CTD-His were inoculated into 500 mL Luria-Bertani medium containing ampicillin (25 mg/mL) or kanamycin (25 mg/mL), incubated at 37°C until the optical density at 600 nm (OD_600_) reached 0.6, and then shaken overnight at 100 rpm at 16°C with 1 mM isopropyl β-D-thiogalactoside. The bacteria were treated with ultrasound, and then the supernatants or precipitates were collected for purification. For baculovirus expression, pFastBac1-mCherry-His-TBK1 or pFastBac1-EGFP-His-SVCV-P was transformed into DH10Bac chemically competent cells (DL1071, WEIDI) to produce a positive recombinant colony, named pFastBac1-mCherry-TBK1-Bac and pFastBac1-EGFP-SVCV-P-Bac. Then, pFastBac1-mCherry-TBK1-Bac and pFastBac1-EGFP-SVCV-P-Bac were transfected into Sf9 cells under the assistance of LipoInsect (c0551, Beyotime) in a T25 flask with Sf-900 II serum-free medium. The cells were cultured at 28˚C for 72 h. The harvested precipitation was dissolved in insect lysing buffer (200 mM Tris-HCl, 150 mM NaCl, 1% NP-40, 1 mM PMSF, pH 8.0). The recombinant protein was purified by Ni-NTA agarose affinity chromatography according to the QIAexpressionist manual (Qiagen). The Bradford method was used to measure the concentration of purified protein (62). For *in vitro* staining of IRF3, purified IRF3 protein was labeled with Alexa Fluor 647 NHS ester, tris (triethylammonium salt) according to the manufacturer’s protocols (Thermo Fisher Scientific).

### Preparation of polyclonal antibodies

Polyclonal antibodies (Abs) were prepared using the recombinant SVCV-P and IRF3 proteins expressed in *E. coli* cells. New Zealand White rabbits, aged 6 weeks and weighing between 1.5 and 2.0 kg, were immunized with either SVCV-P or IRF3 protein (0.5 mg/kg) three times, followed by the method as previously described (63). Antisera were collected following the final immunization when Ab titers reached above 1:10,000 as determined by microplate-based ELISA. The specificity of the Abs (anti-SVCV-P and anti-IRF3) was further validated through Western blot analysis. For triple staining of SVCV-P-TBK1-IRF3, rabbit anti-SVCV-P Ab was conjugated with AlexaFluor 647, and rabbit anti-IRF3 Ab was conjugated with AlexaFluor 555 by Bioss Biotechnology Co., Ltd (Beijing, China).

### *In vitro* phase separation assay

For the SVCV-P *in vitro* phase separation assay, experiments were performed in LLPS storage buffer (30 mM Tris-HCl, 200 mM NaCl, 5% PEG8000, pH 7.5) using the indicated SVCV-P concentrations. Phase separation assays involving SVCV-P/TBK1, TBK1/IRF3, or SVCV-P/TBK1/IRF3 were similarly conducted in the LLPS storage buffer. All assays were carried out in glass-bottom dishes sealed with optically clear adhesive film to prevent evaporation. Phase separation was observed under a confocal laser scanning microscope (Olympus FV3000) with a 63× oil immersion lens.

### Fluorescence recovery after photobleaching

FRAP was performed using a confocal laser scanning microscope (Olympus FV3000) equipped with a 63× oil immersion lens. For *in vitro* cellular experiments, HEK293T cells transfected with the indicated plasmids were placed in a live-cell imaging chamber at 37°C. Regions of interest (ROIs) within puncta-positive cells were defined using the confocal software and bleached with the 488 nm laser at 100% power. Time-lapse images were acquired every 3 s for 8 min post-bleaching. ROI fluorescence intensities were corrected using unbleached control regions and normalized to their respective pre-bleached intensities. For *in vitro* FRAP assays on droplets, a single bleach pulse was applied using the 488 nm laser at 50% power; data were collected from at least five samples. Recovery curves were generated using GraphPad Prism 8 software.

### Attenuated total reflection Fourier-transform infrared spectroscopy

ATR-FTIR measurements were performed using a Nicolet iS50 spectrophotometer (Thermo Fisher Scientific, USA) to characterize the secondary structural changes of SVCV-P-TBK1-IRF3 condensates. The samples collected at 0 h and 24 h were applied onto the diamond ATR crystal, and spectra were acquired from 1,500 to 1,700 cm⁻¹ with a resolution of 4 cm⁻¹. All spectra were baseline-corrected and area-normalized within the amide I region (1,600–1,700 cm⁻¹) before analysis. Peak profiles were analyzed using Origin 2025b software to monitor shifts in the amide I band indicative of β-sheet formation.

### MST assay

MST assays were performed as previously described (64). Binding affinities of purified SVCV-P or IRF3 for mCherry-tagged TBK1 protein were measured using a Monolith NT.115 instrument (NanoTemper Technologies). For each assay, the labeled protein was incubated with an equal volume of unlabeled ligand at 16 different concentrations in the same buffer used for protein storage, at room temperature for 10 min. Samples were then loaded into standard-treated capillaries (NanoTemper Technologies) and measured using 40% light-emitting diode and medium MST power. Each assay was repeated three to five times. Dissociation constants (*K*_d_ values) were calculated using MO.Affinity Analysis software. Final binding curves were plotted using GraphPad Prism 8.0, and the given *K*_d_ values were calculated with 95% confidence levels.

### Luciferase activity assay

EPC cells were seeded in 24-well plates and incubated overnight. Cells were co-transfected with various plasmid combinations (pCMV-P and variants), IFNφ1-pro (250 ng/mL), IFNφ3-pro (250 ng/mL), and the pRL-TK Renilla luciferase internal control plasmid (25 ng/mL) using FishTrans transfection reagent (MeiSenTe Biotechnology). The empty vector pCMV-flag was included to maintain equal total DNA amounts per well. Poly(I:C) transfection or SVCV infection was carried out 24 h prior to cell harvest. At 48 h post-transfection, cells were washed thrice with PBS, lysed, and luciferase activity was measured using the Dual-Luciferase Reporter Assay System (Promega), following the manufacturer’s protocol. Relative luciferase activity units were calculated as the ratio of firefly luciferase activity to Renilla luciferase activity, as previously described (61). All experiments were performed independently at least three times.

### IF staining

HEK293T, EPC, or ZF4 cells were seeded onto coverslips in a 12-well plate and then infected with 100 TCID_50_ SVCV (5 × 10^6^ TCID_50_/mL) or transfected with the indicated plasmids. After 24 h, cells were washed thrice with PBS and fixed with 4% paraformaldehyde for 10 min and permeabilized with 0.25% Triton X-100 at room temperature for 10 min. Subsequently, the cells were blocked with 2% bovine serum albumin and incubated with primary antibodies for 2 h. After rinsing, cells were incubated with the corresponding secondary antibodies (Sangon Biotech) according to the manufacturer’s instructions. After another round of PBS washing, nuclei were stained with 4′,6-diamidino-2-phenylindole (DAPI) (100 ng/mL; Sigma-Aldrich) at 37°C for 10 min. Fluorescence images were acquired using a laser scanning confocal microscope (Olympus FV3000) with a 63× oil immersion objective.

### RNA interference experiment

Four short hairpin RNAs (shRNAs; designated as shP#1, shP#2, shP#3, and shP#4) targeting the SVCV-P mRNA were designed using BLOCK-iT RNAi Designer and cloned into the pLKO.1-TRC cloning vector. Three SVCV-P mRNA-targeting siRNAs (designated as siP#1, siP#2, and siP#3) and a control siRNA were designed and synthesized by Generay Biotech (Shanghai, China) as reported earlier (65), with the following sequences: siP-1 (5′-CGGAUAUGACAUUGAUCUA-3′), siP-2 (5′-CAUCCGAGAUUUAUUCAUA-3′), and siP-3 (5′-GACAGUCAAUUGGGAAGA A-3′). EPC cells were seeded in 6-well plates overnight and then transfected with either the indicated plasmids (1.5 µg) or siRNAs (100 nM) using FishTrans transfection reagent (MeiSenTe Biotechnology) according to the manufacturer’s protocol. At 24 h post-transfection, cells were infected with 100 TCID_50_ of SVCV (5 × 10^6^ TCID_50_/mL) for knockdown and functional evaluation. Knockdown efficiency for SVCV-P expression was assessed by immunoblotting with anti-SVCV-P Ab and measuring IFN-φ1 or IFN-φ3 promoter reporter activity, as described above.

### Preparation of capped mRNA

For mRNA synthesis, cDNA sequences encoding SVCV-P FL, SVCV-P∆IDR, SVCV-P_ΔIDR_-FUS_IDR_, and SVCV-P-P_ΔIDR_-IRF3_IDR_ were cloned into the pBluescriptII vector using primers listed in Table S1. The capped mRNA was synthesized using the mMESSAGE Kit (Ambion), purified with the MEGAclear Kit (Invitrogen), and then dissolved in DEPC-treated water.

### Functional evaluation in zebrafish embryos

The repressive function mediated by SVCV-P phase separation was examined *in vivo* using zebrafish embryos. For this procedure, one-cell stage embryos were co-microinjected with siP#2 (100 nM), capped mRNAs encoding SVCV-P FL, SVCV-P∆IDR or SVCV-P_ΔIDR_-FUS_IDR_ or SVCV-P-P_ΔIDR_-IRF3_IDR_) (150 pg/embryo), IFNφ1-pro (100 pg/embryo), and pRL-TK (10 pg/embryo) reporter vectors. At 12 h post-injection (hpi), the embryos were inoculated with either mock PBS (control) or SVCV (2 nL/embryo; 5 × 10^6^ TCID_50_/mL), followed by measurement of luciferase activities at 24 hpi. Concurrently, survival analysis was conducted. Embryos were microinjected with siP#2 and the indicated mRNAs prior to SVCV infection as detailed above. Survival rates were continuously calculated for up to 60 hpi, followed by statistical analysis.

### Bioinformatics analysis

The domain structures of SVCV-P, IRF3, and TBK1 were analyzed using SWISS-MODEL (66), and the secondary structures of the SVCV-P, IRF3, and TBK1 proteins were predicted using Expasy (67). The tertiary structural figures were reviewed by the AlphaFold3 method (68) and colored using PyMOL software (69).

### Statistical analysis

All data are presented as means ± standard deviations (SD). Normal distribution was confirmed prior to conducting comparisons between two groups using a two-tailed Student’s t-test. For experiments involving more than two samples, one-way analysis of variance was performed. Survival curve differences were assessed using the log-rank test. Statistical significance was defined as *P* < 0.05 (^*^), *P* < 0.01 (^**^), and *P* < 0.001 (^***^), as indicated in the figures. Each experiment was independently replicated at least three times.

## Data Availability

The authors confirm that the data supporting the findings of this study are available within the article.

## SUPPLEMENTAL MATERIAL

Supplemental material jvi.01387-25-s0001.docx Fig. S1 and S2; Table S1.

ASM does not own the copyrights to Supplemental Material that may be linked to, or accessed through, an article. The authors have granted ASM a non-exclusive, world-wide license to publish the Supplemental Material files. Please contact the corresponding author directly for reuse.
