# Supplementary material for: SVCV phosphoprotein hijacks phase separation to immobilize the IRF3-TBK1 signaling axis and suppress interferon antiviral immunity
Source: J Virol. 2025 Dec 9;100(1):e01387-25. doi: 10.1128/jvi.01387-25 (PMC12817906; doi:10.1128/jvi.01387-25)
Supplement: Supplemental material — Fig. S1 and S2; Table S1. [file jvi.01387-25-s0001.docx]

**Supplemental information**

**SVCV phosphoprotein hijacks phase separation to immobilize the IRF3-TBK1 signaling axis and suppress interferon antiviral immunity**

Yueyi Wang^1^, Fengyun Wu^1^, Dongdong Fan^1^, Aifu Lin^1^, Lixin Xiang^1*^, Ye Chen^1*^, Jianzhong Shao^1,2*^,

^1^College of Life Sciences, Key Laboratory of Cell and Molecular Intelligent Design and Development of Zhejiang Province, Division of Medical Genetics and Genomics, the Children’s Hospital, Zhejiang University School of Medicine, Zhejiang University, Hangzhou, China; ^2^Laboratory for Marine Biology and Biotechnology, Qingdao Marine Science and Technology Center, Qingdao 266071, China

^*^Corresponding authors: shaojz@zju.edu.cn (JZS), yechency@zju.edu.cn (YC) and xianglx@zju.edu.cn (LXX).

Address correspondence and reprint requests to Prof. Jianzhong Shao, Prof. Ye Chen and Assoc. Prof. Li-xin Xiang, College of Life Sciences, Zhejiang University, 866 YuHangTang Road, Hangzhou 310058, China. Tel.: +86 (571) 88206582; Fax: +86 (571) 88206582. E-mail addresses: shaojz@zju.edu.cn (JZS), yechency@zju.edu.cn (YC) and xianglx@zju.edu.cn (LXX)

**Running title：New insights into the role of phase separation in SVCV immune evasion**

**SUPPLEMENTAL FIGURE 1**


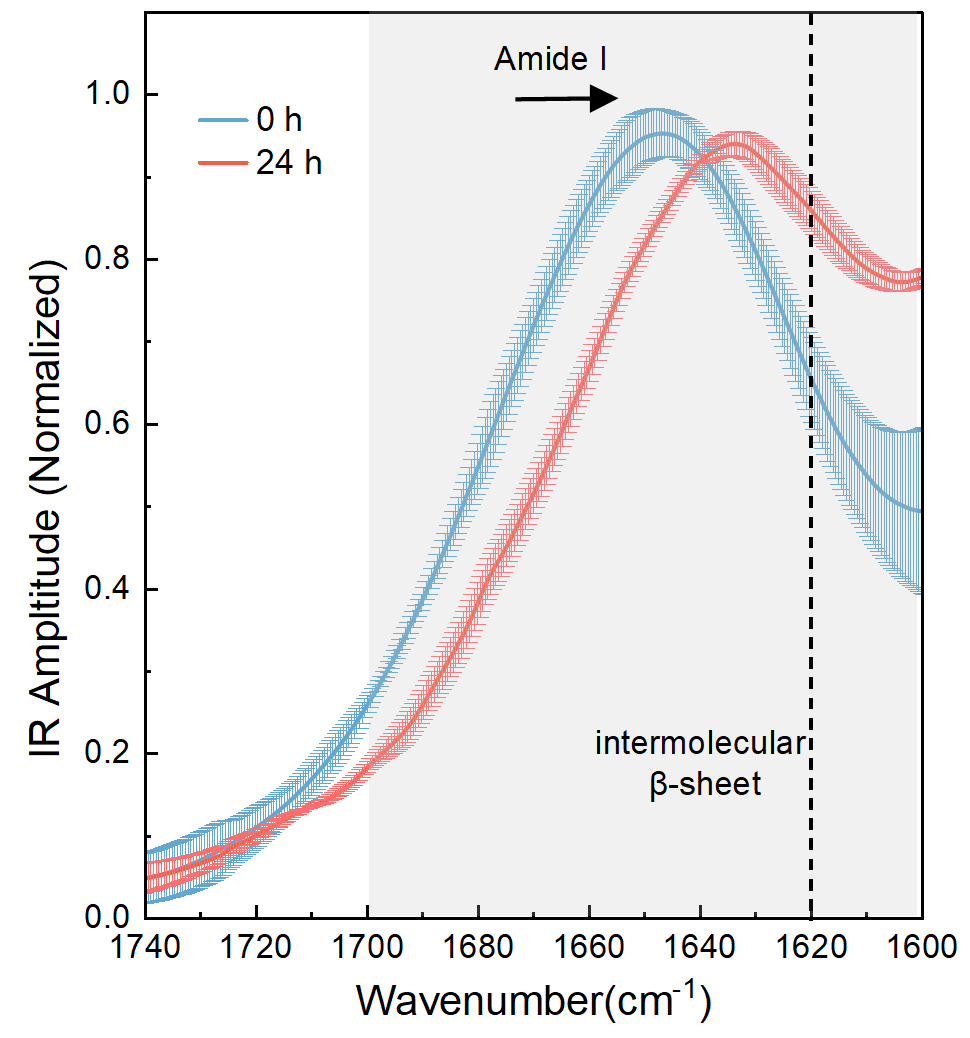


**Supplementary Figure 1.** Attenuated Total Reflection Fourier-Transform Infrared Spectroscopy (ATR-FTIR) spectra of SVCV-P-TBK1-IRF3 condensates at 0 h (blue) and 24 h (red) showing the amide I band (1600-1700 cm⁻¹). A shift of the amide I peak toward 1630 cm⁻¹ and increased absorption in the β-sheet region indicate the formation of intermolecular hydrogen bonds and a transition from liquid-like to more solid-like condensates during incubation.

**SUPPLEMENTAL FIGURE 2**
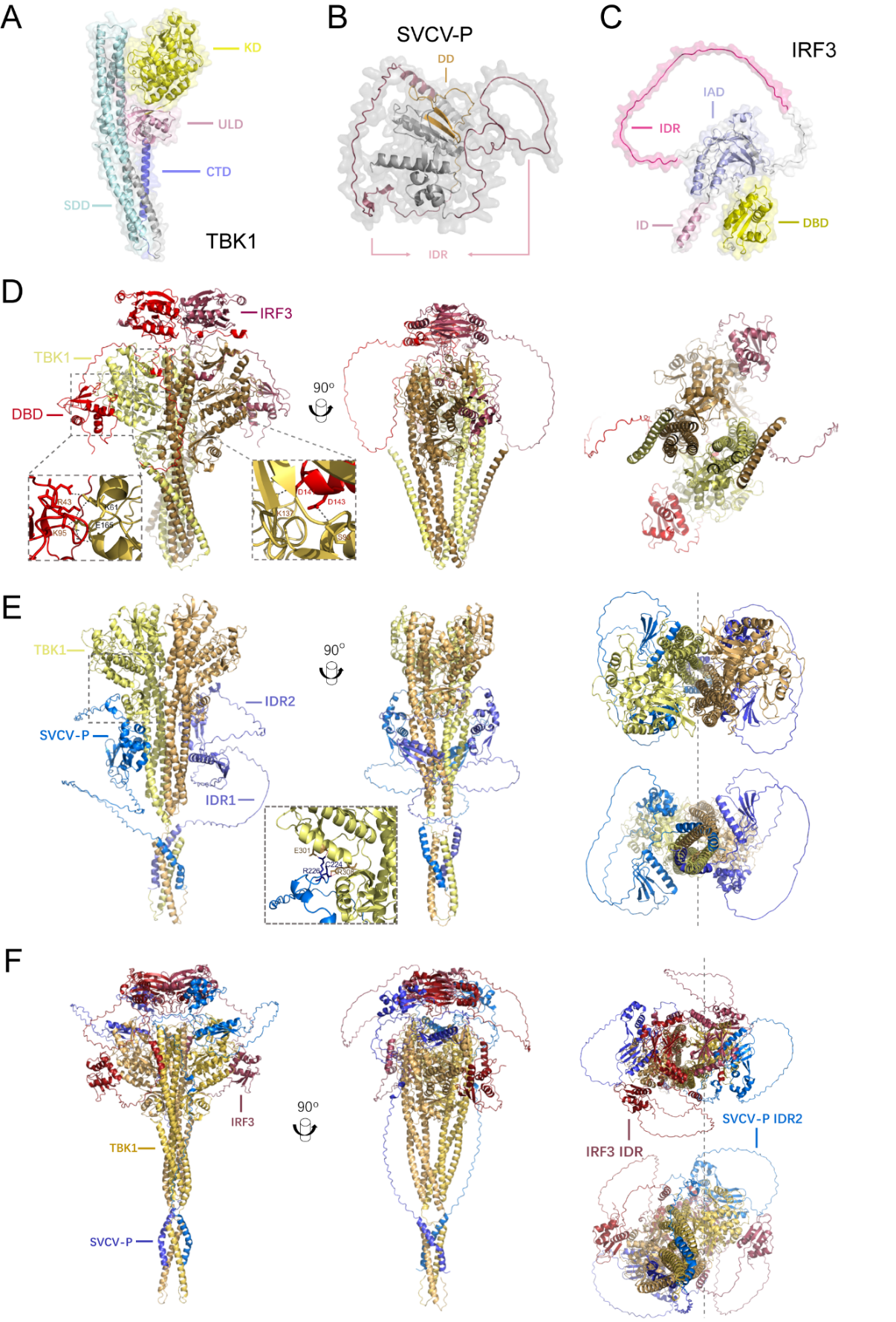


**Supplementary Figure 2.** Predicted structures of SVCV-P, TBK1, IRF3, and associated complexes using AlphaFold3. (A-C) Overall individual structures of TBK1, SVCV-P and IRF3. (D) Overall structure of the TBK1 dimer in complex with two IRF3 molecules, showing a detailed view of the IRF3-DBD with TBK1-KD interactions. (E) Overall structure of the TBK1 dimer complexed with two SVCV-P molecules, highlighting interactions between the SVCV-P-IDR2 and TBK1-KD. (F) Tertiary structure of the SVCV-P/TBK1/IRF3 ternary complex.

**SUPPLEMENTAL TABLE S1. Primers used in the experiments**

| **Name** | **Sequence (5’→3’)** | **Name** | **Sequence (5’→3’)** | **Application** |
| --- | --- | --- | --- | --- |
| P-28a F | CAGCAAATGGGTCGCGGATCCATGTCTCTACATTCGAAATT | TBK1-pFast-F | GACTCTAGACTGCAGCTCGAGATGCAGAGTACGGCCAATTA | Plasmid construction |
| P-28a R | TTGTCGACGGAGCTCGAATTCCAACCTATATTTTTGATACA | TBK1-pFast-R | AGGACCCTGGAACAGAACTTCCAGCATCCGCTCCACTGTCCTCA |  |
| P-6Myc-F | CTTGGTACCGAGCTCGGATCCATGTCTCTACATTCGAAATT | mCherry- pFast-F | CTGGAAGTTCTGTTCCAGGGTCCTATGGCCTCCTCCGAGAACGT |  |
| P-6Myc-R | CCACACTGGACTAGTGGATCCCAACCTATATTTTTGATACA | mCherry- pFast-R | CTAGTACTTCTCGACTCAATGGTGATGGTGATGGTGATGATGCTACAGGAACAGGTGGT |  |
| P-EGFP-28a-F | CAGCAAATGGGTCGCGGATCCATGTCTCTACATTCGAAATT | TBK1 RFP F | TCAGATCTCGAGCTCAAGCTTATGCAGAGTACGGCCAATTA |  |
| P-EGFP-28a-R | TTGTCGACGGAGCTCGAATTCCTTGTACAGCTCGTCCATGC | TBK1 RFP R | CGACTGCAGAATTCGAAGCTTCATCCGCTCCACTGTCCTCA |  |
| P-pFast-F | CGCGGATCCAGATCTGAATTCATGTCTCTACATTCGAAATT | TBK1 flag F1 | TCCCCCGGGCTGCAGGAATTCATGCAGAGTACGGCCAATTA |  |
| P-pFast-R | CAGTCTAGAGTCGACGAATTCCAACCTATATTTTTGATACA | TBK1 flag R1 | TCTTCGCCCTTAGACACCATGGCGGTCATCCGCTCCACTGTCCTCA |  |
| P IDR F1 | TCAGATCTCGAGCTCAAGCTTATGTCTCTACATTCGAAATT | BFP F1 | TGAGGACAGTGGAGCGGATGACCGCCATGGTGTCTAAGGGCGAAGA |  |
| P IDR R1 | ACGTTACCGAGGCCGTTATACTCAGACGAGTCCTCCTCAA | BFP R1 | GATAAGCTTGATATCGAATTCATTAAGCTTGTGCCCCAGTT |  |
| P IDR F2 | TTGAGGAGGACTCGTCTGAGTATAACGGCCTCGGTAACGT | TBK1-ΔKD-F1 | TCCCCCGGGCTGCAGGAATTCATGCAGAGTACGGCCAATTACCTGTGG |  |
| P IDR R2 | ATCTCCCATAATCCCTCTGGTGGCTTGTGGGGTAAGAACA | TBK1-ΔKD-F1-1 | GTACGGCCAATTACCTGTGGGTCTTCAGTCTGCAGCAGGC |  |
| P IDR F3 | TGTTCTTACCCCACAAGCCACCAGAGGGATTATGGGAGAT | TBK1-ΔKD-R | GATAAGCTTGATATCGAATTCCATCCGCTCCACTGTCCTCA |  |
| P IDR R3 | CGACTGCAGAATTCGAAGCTTCAACCTATATTTTTGATACA | TBK1-ΔULD-R1 | TGTACTTTGGGTGGGCTGGGGATGCGGTGTAAGATGTCGC |  |
| P sh1-F | CCGGGGATGAAGATGAAGAAGATCACTCGAGTGATCTTCTTCATCTTCATCCTTTTTG | TBK1-ΔULD-F2 | GCGACATCTTACACCGCATCCCCAGCCCACCCAAAGTACA |  |
| P sh1-R | AATTCAAAAAGGATGAAGATGAAGAAGATCACTCGAGTGATCTTCTTCATCTTCATCC | TBK1-ΔSDD-R1 | AACTGACCAGTCAGAGACAGTACTTTGGGTGGGCTGGGGT |  |
| P sh2-F | CCGGGGAGGTACATATGTTCGTTATCTCGAGATAACGAACATATGTACCTCCTTTTTG | TBK1-ΔSDD-F2 | ACCCCAGCCCACCCAAAGTACTGTCTCTGACTGGTCAGTT |  |
| P sh2-R | AATTCAAAAAGGAGGTACATATGTTCGTTATCTCGAGATAACGAACATATGTACCTCC | TBK1-ΔCTD-R1 | ACCGTCAGTGAGCCGAATCTACCAGACGCCATCGGGACCA |  |
| P sh3-F | CCGGGCACTGTAATCACAGCCAAACCTCGAGGTTTGGCTGTGATTACAGTGCTTTTTG | TBK1-ΔCTD-F2 | TGGTCCCGATGGCGTCTGGTAGATTCGGCTCACTGACGGT |  |
| P sh3-R | AATTCAAAAAGCACTGTAATCACAGCCAAACCTCGAGGTTTGGCTGTGATTACAGTGC | ∆SDD-pFast F | GACTCTAGACTGCAGCTCGAGATGCAGAGTACGGCCAATTA |  |
| P sh4-F | CCGGGGGATCAAACTGAGGAGATTGCTCGAGCAATCTCCTCAGTTTGATCCCTTTTTG | ∆SDD-pFast R | TCCTCGCCCTTGCTCACCATCATCCGCTCCACTGTCCTCA |  |
| P sh4-R | AATTCAAAAAGGGATCAAACTGAGGAGATTGCTCGAGCAATCTCCTCAGTTTGATCCC | ∆SDD mCherry pfast F | TGAGGACAGTGGAGCGGATGATGGTGAGCAAGGGCGAGGA |  |
| IRF3 RFP F | TCGAGCTCAAGCTTCGAATTCTGATGACTCAAGCAAAACCGCT | ∆SDD mCherry pfast R1 | ATGGTGATGGTGATGGTGATGATGCTTGTACAGCTCGTCCATGC |  |
| IRF3 RFP R | GGATCCCGGGCCCGCGGTACCGTGCAGAGCTCCATCATTTGCT | ∆SDD mCherry pfast R2 | CTAGTACTTCTCGACAAGCTTTCAATGGTGATGGTGATGGTGATGATG |  |
| IRF3 GFP F | TCAGATCTCGAGCTCAAGCTTATGACTCAAGCAAAACCGCT | ∆SDD-RFP-flag F | TCCCCCGGGCTGCAGGAATTCATGCAGAGTACGGCCAATTA |  |
| IRF3 GFP R | CGACTGCAGAATTCGAAGCTTGCAGAGCTCCATCATTTGCT | ∆SDD-RFP-flag R | ATCTTGTCCAGGTTGGCCATCATCCGCTCCACTGTCCTCA |  |
| IRF3-28a F | CAGCAAATGGGTCGCGGATCCATGACTCAAGCAAAACCGCT | ∆SDD Nano F | TGAGGACAGTGGAGCGGATGATGGCCAACCTGGACAAGAT |  |
| IRF3-28a R | TTGTCGACGGAGCTCGAATTCGCAGAGCTCCATCATTTGCT | ∆SDD Nano R | GATAAGCTTGATATCGAATTCGCTCTGCTGAATGGCGATGC |  |
| IRF3-3.1HA-F | GCTAGCGCCACCATGGCGGCCGCAATGACTCAAGCAAAACCGCT | IRF3-3.1HA-R | GTATGGGTAACCGGTAAGCTTGCAGAGCTCCATCATTTGCT |  |
| P-pblue F | GATAAGCTTGATATCGAATTCATGTCTCTACATTCGAAATT | P-pblue R | CGCTCTAGAACTAGTGGATCCCTACAACCTATATTTTTGAT |  |
| P-pblue-Fus R1 | TGGGTATAATCGTTTGAGGCCTCAGACGAGTCCTCCTCAA | FUS F | TTGAGGAGGACTCGTCTGAGGCCTCAAACGATTATACCCA |  |
| FUS R | ACGTTACCGAGGCCGTTATAGCCTCCACGGTCCTGCTGTC | P-FUS XIA F | GACAGCAGGACCGTGGAGGCTATAACGGCCTCGGTAACGT |  |
| P-pblue-IRF3 R | GAGGATATGGCTTCAATAGGCTCAGACGAGTCCTCCTCAA | IRF3 IDR F | TTGAGGAGGACTCGTCTGAGCCTATTGAAGCCATATCCTC |  |
| IRF3 IDR R | ACGTTACCGAGGCCGTTATAAGTGTGATCAGGTACAGCTC | P-IRF3 XIA F | GAGCTGTACCTGATCACACTTATAACGGCCTCGGTAACGT |  |
| siP-1 sense | CGGAUAUGACAUUGAUCUA | siP-1 anti-s | UAGAUCAAUGUCAUAUCCG | RNA interference |
| siP-2 sense | CAUCCGAGAUUUAUUCAUA | siP-2 anti-s | UAUGAAUAAAUCUCGGAUG |  |
| siP-3 sense | GACAGUCAAUUGGGAAGAA | siP-3 anti-s | UUCUUCCCAAUUGACUGUC |  |
| M qRT F | TACTCCTCCCACTTACGA | IFNφ1 qRT F | CAAGTCAAAGGTGGAGGACC |  |
| M qRT R | CAAGAGTCCGAGAAGGTC | IFNφ1 qRT R | TCTTGCCACACATTCTTTGAGG |  |
| N qRT F | GCGGTTTTCTGTATGTGTCTC | IFNφ3 qRT F | CTCTAATCAGAAAACCGGTG |  |
| N qRT R | CTCTGCCAAATCACCATACTC | IFNφ3 qRT R | CTCATCCTCATAATACATTGGTT |  |
| G qRT F | CGACCTGGATTAGACTTG | IFN-EPC F | ATGAAAACTCAAATGTGGACGTA | Real-time PCR |
| G qRT R | AATGTTCCGTTTCTCACT | IFN-EPC R | GATAGTTTCCACCCTTTCCTTAA |  |
| EPC-IFN qRT F | ATGAAAACTCAAATGTGGACGTA | IFNφ1 qRT F | CAAGTCAAAGGTGGAGGACC |  |
| EPC-IFN qRT R | GATAGTTTCCACCCTTTCCTTAA | IFNφ1 qRT R | TCTTGCCACACATTCTTTGAGG |  |

F, forward primer; R, reverse primer.
